# Pre-seeding diabatic anchors for reproducible direct-dynamics quantum photodissociation: an 8-state full-dimensional DD-vMCG study of CH_2_OO

**DOI:** 10.1039/d6cp02996e

**Published:** 2026-09-29

**Authors:** Kevin Ma, Graham A. Worth

**Affiliations:** a Department of Chemistry, University College London London WC1H 0AJ UK g.a.worth@ucl.ac.uk

## Abstract

Direct-dynamics quantum simulations construct a potential-energy representation from electronic-structure data collected at selected nuclear geometries. When these data are accumulated during propagation, the resulting quasi-diabatic potentials can depend on the geometries encountered early in the calculation. We investigate whether supplying reference geometries in advance reduces this sensitivity in the photodissociation of CH_2_OO. A database containing the Franck–Condon reference and 97 additional entries along the mass- and frequency-weighted *Q*_3_ coordinate (dominated by O–O stretching) is compared with one initially confined to the Franck–Condon region, using the same direct-dynamics variational multi-configurational Gaussian (DD-vMCG) method. Both calculations include eight electronic states and all nine vibrational degrees of freedom at the SA-CASSCF(10e,8o)/6-31G(d,p) level, with production databases of approximately 5000 points. Along the sampled path, informed sampling produces smoother diagonal quasi-diabatic potentials and more clearly ordered changes in electronic-state composition. Both workflows show O–O extension, but the informed-sampling calculation retains population in the initially excited adiabatic state, *S*_2_, for longer before rapid multistate redistribution. These results identify database initialisation as a practical influence on the Hamiltonian representation and its predicted population dynamics. The state correlations support provisional assignments of the photodissociation channels, while quantitative branching ratios require further convergence and channel-resolved flux analysis.

## Abstract 2

Direct-dynamics quantum simulations construct a potential-energy representation from electronic-structure data collected at selected nuclear geometries.

## Graphical abstract

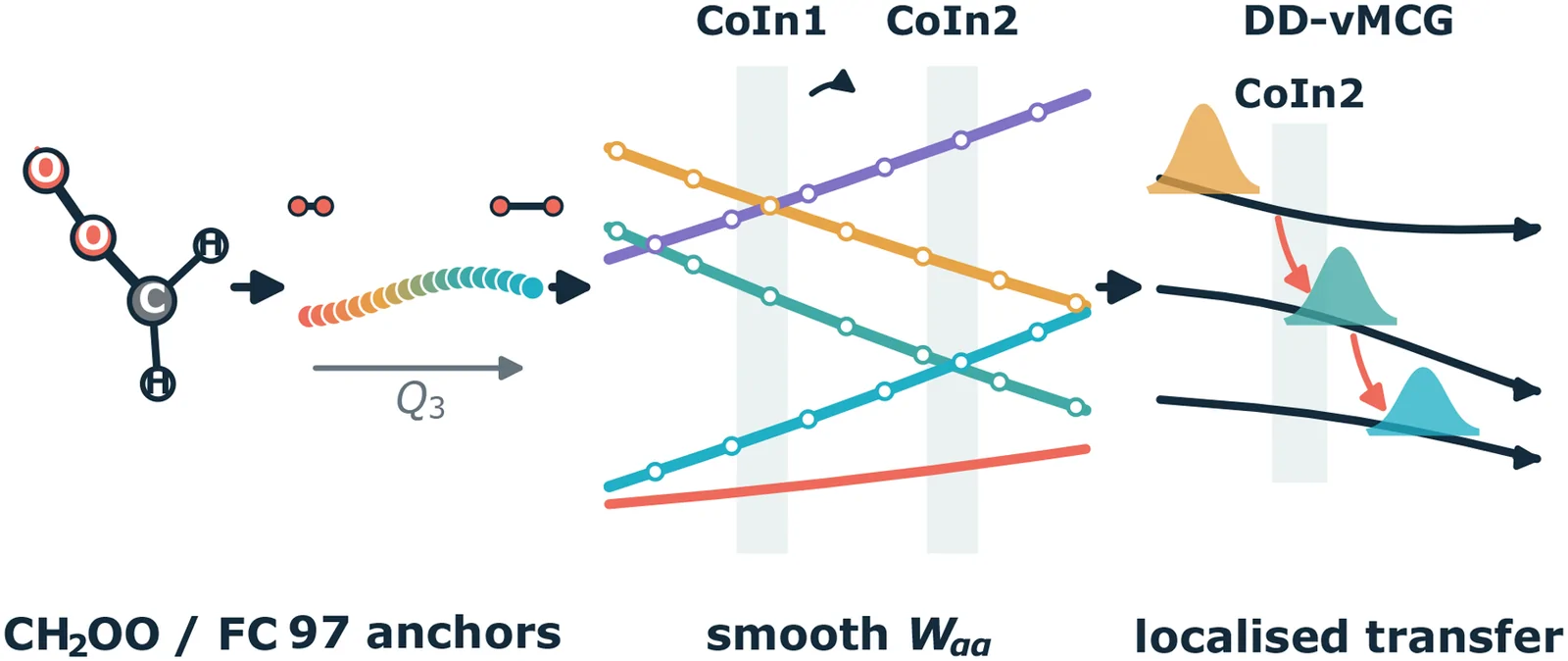

## 1 Introduction

Conical intersections (CIs) provide efficient pathways for nonadiabatic population transfer and internal conversion in polyatomic molecules and can shape photochemical branching on femtosecond timescales.^1–6^ Simulating these processes requires a coupled treatment of nuclear motion and electronic states beyond the Born–Oppenheimer approximation.^1,7,8^ Two challenges are particularly relevant to reactive photochemistry: constructing global multistate potential-energy surfaces (PESs) becomes increasingly demanding as the number of nuclear degrees of freedom and coupled electronic states grows, while the adiabatic representation becomes numerically challenging near degeneracies because derivative couplings can be sharply peaked or formally singular.^1,8^

Nonadiabatic dynamics methods balance their treatment of nuclear and electronic motion, computational cost and accessible system size in different ways. Trajectory-based approaches such as trajectory surface hopping and trajectory-guided Gaussian-basis methods such as *ab initio* multiple spawning have enabled full-dimensional on-the-fly studies of many photochemical systems by evaluating electronic-structure quantities at dynamically sampled nuclear configurations.^3,9–12^ By avoiding the prior construction of global coupled surfaces, these established methods provide practical access to molecular systems and timescales that remain difficult for grid-based quantum dynamics. The appropriate description depends on the scientific question: methods that explicitly represent the nuclear wavefunction can describe coherence, interference and tunneling within that representation, but introduce their own basis-convergence and computational requirements.^6,10–12^ Grid-based and other precomputed-surface wavepacket calculations also require coupled PESs whose construction becomes progressively more demanding with increasing dimensionality, state number and dissociation range.^13–15^

The direct-dynamics variational multi-configuration Gaussian method (DD-vMCG) combines explicit nuclear-wavefunction propagation with on-the-fly electronic-structure evaluation.^16–19^ In vMCG, the nuclear wavefunction is expanded in a moving basis of coupled Gaussian wavepackets (GWPs), and the equations of motion are derived variationally.^20^ In the direct-dynamics formulation, local harmonic expansions are constructed from electronic-structure calculations requested at geometries sampled by the evolving GWP basis, while database reuse and modified Shepard interpolation reduce repeated quantum-chemistry evaluations.^17,21^ By avoiding a global PES fit in advance, this formulation can be applied to photodissociation problems in which the wavepacket rapidly leaves the Franck–Condon region and samples several coupled nuclear modes. Its accuracy nevertheless remains dependent on convergence of the Gaussian basis and on the coverage and quality of the electronic-structure database.

An additional challenge concerns the representation of the coupled electronic Hamiltonian near CIs. In an adiabatic basis, derivative couplings enter the nuclear kinetic-energy operator and can become large near a degeneracy, while the dominant electronic character of energy-ordered states may change abruptly.^1,8^ A diabatic or quasi-diabatic transformation transfers much of this coupling from derivative terms to off-diagonal potential-matrix elements, yielding a representation that is generally smoother through the interaction region. A globally strict diabatic basis, however, is generally unavailable for polyatomic molecules over an extended region of configuration space.^1,8^ The DD-vMCG implementation used here therefore adopts propagation diabatisation: the adiabatic-to-diabatic transformation (ADT) matrix is propagated along nuclear displacements sampled by the GWP basis using nonadiabatic coupling vectors, with predictor-corrector procedures that maintain phase continuity and regularise updates across detected seam crossings.^22^ Because practical calculations retain a finite electronic manifold, the resulting representation remains quasi-diabatic and may retain path dependence when the selected states do not form an effectively isolated subspace.^8,22^

Here we examine a distinct practical source of initialisation sensitivity in multistate DD-vMCG: sampling-history dependence in the growing electronic-structure database. In a dark-start workflow, the database is initially empty or confined to the Franck–Condon region, and exploratory propagation determines which geometries first become reference points for interpolation and diabatisation.^17,22^ If the evolving basis reaches a dense multistate interaction region before reference geometries sample both sides of it, the local quasi-diabatic gauge may become conditioned by the order and spatial distribution of the first database insertions. We refer to this algorithmic initialisation effect as database sampling-history dependence. It is distinct from the formal path dependence of finite-state ADT propagation. The present study tests whether such dependence persists after subsequent database growth and whether it is expressed through excess curvature in the diagonal diabatic potentials, less coherent electronic-character tracking and less well-defined asymptotic state correlation.

We introduce an informed-sampling protocol designed to reduce this database-history dependence. Before exploratory propagation, electronic-structure data are calculated at geometries covering an anticipated reaction path and relevant interstate-interaction regions and inserted into the database. We refer to these preselected geometries as seed geometries and to their stored records as seed entries. Their potentials are diabatised using the same predictor-corrected procedure applied during subsequent database construction. These entries act as diabatic anchors, extending the initial reference coverage without changing the vMCG ansatz or the ADT construction used in the subsequent dynamics. The workflow is not restricted to dissociation: geometries along an isomerisation path could also supply seed entries, although their effectiveness would depend on how well they cover the excited-state regions subsequently sampled.

We test the protocol on CH_2_OO, the simplest Criegee intermediate. Criegee intermediates are atmospheric carbonyl oxides formed during alkene ozonolysis, and CH_2_OO has been studied extensively in near-UV nonadiabatic photochemistry because O–O dissociation involves several low-lying singlet states and multiple near-degeneracy regions.^23–33^ Previous theoretical studies have characterised its absorption spectrum, dissociation pathways and product-channel correlations using reduced-dimensional quantum dynamics, fitted quasi-diabatic models and trajectory-based direct dynamics.^27,28,30–33^ The present study does not aim to determine converged branching ratios for CH_2_OO. Instead, its aim is twofold. First, the eight-state, full-dimensional (9D) DD-vMCG calculation at the state-averaged complete active space self-consistent field level [SA-CASSCF(10e,8o)/6-31G(d,p)] tests the application of our diabatisation protocol to a dense singlet manifold with multiple interaction regions. Second, the informed-sampling comparison examines how the initial reference coverage affects the quasi-diabatic Hamiltonian, electronic-state correlation and propagated populations after subsequent database growth. The work is thus concerned with the integration and assessment of initial sampling within this workflow, rather than the general idea of sampling important configurations.

Our broader aim is to make the preparation of such calculations more systematic by specifying how reference geometries are diabatised and incorporated into subsequent database growth. The recent cyclobutanone prediction challenge,^34^ in which different methods were used to simulate one observable, illustrated the difficulty of isolating individual methodological choices when electronic-structure treatment, initial preparation, dynamics implementation and the propagating Hamiltonians differ.

The paper is organised as follows. Section 2 summarises the vMCG/DD-vMCG framework and the predictor-corrected propagation-diabatisation scheme.^16,17,22^ Section 3 describes the electronic-structure level, coordinate system, initial wavepacket, database construction and informed-sampling protocol.^35–38^ Section 4 compares the workflows through three diagnostics: the smoothness of one-dimensional diabatic Hamiltonian cuts, the coherence of adiabatic-character tracking along O–O dissociation and differences in full-dimensional wavepacket observables. It then defines the limits of the resulting channel analysis and the convergence and channel-resolved flux calculations required before quantitative product yields can be assigned.

## 2 Theory

### 2.1 The variational multiconfigurational Gaussian method

Only the elements of vMCG required for the present discussion are summarised here; full derivations are available elsewhere.^16–19,39^ We use ***q*** for the collective electronic coordinates and ***Q*** for the collective nuclear coordinates. The molecular time-dependent Schrödinger equation (TDSE) is1
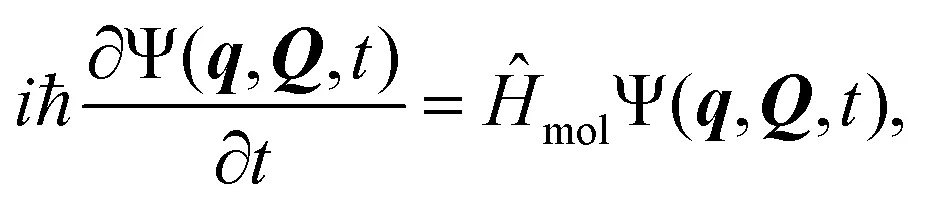
with

2 *Ĥ*_mol_ = *T̂*_*n*_(***Q***) + *Ĥ*_el_(***q***;***Q***),

where *T̂*_*n*_ is the nuclear kinetic-energy operator and *Ĥ*_el_ is the clamped-nuclei electronic Hamiltonian. Projection onto a finite electronic subspace gives the Born representation3
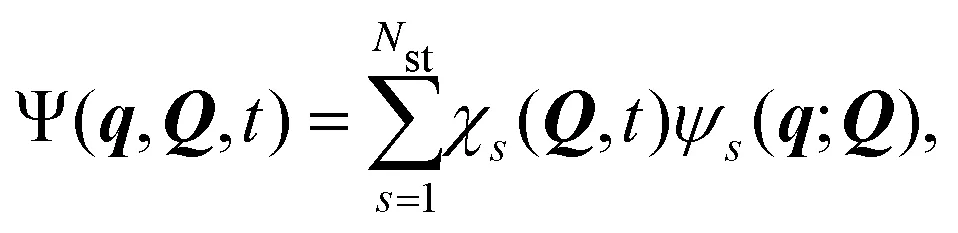
where *ψ*_*s*_(***q***;***Q***) is an electronic basis function and *χ*_*s*_(***Q***,*t*) is its nuclear wavefunction component. If the electronic basis functions are eigenfunctions of the electronic Hamiltonian at ***Q***, after integration over the electronic coordinates the TDSE becomes4
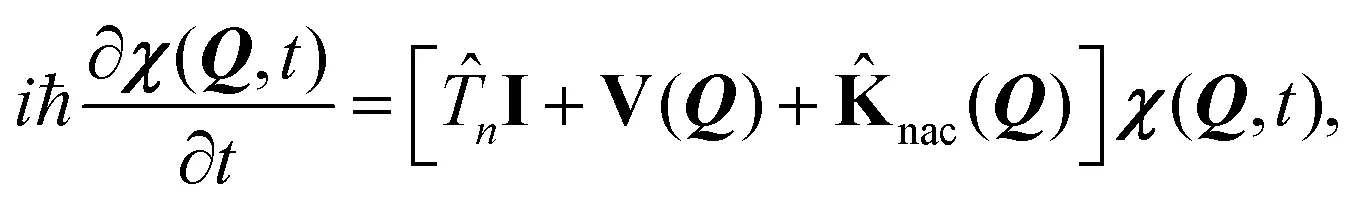
where **V**(***Q***) is the electronic potential matrix and ***K̂***_nac_(***Q***) contains the first- and second-order derivative-coupling operators generated by the nuclear-coordinate dependence of the electronic basis. These terms, with inverse dependency on energy differences, can become large in an adiabatic representation near a conical intersection. A strictly diabatic representation, where one can be defined, removes these operators, whereas a quasi-diabatic transformation seeks to reduce them and to represent the dominant interstate coupling through off-diagonal potential-matrix elements.^22,40,41^ The calculations below use the working quasi-diabatic Hamiltonian *T̂*_*n*_**I** + *Ŵ*(***Q***), neglecting residual derivative-coupling operators in the propagated quasi-diabatic basis. The nuclear coordinates are rectilinear mass-frequency weighted normal modes, ***Q*** = (*q*_1_,…,*q*_*f*_), for which *T̂*_*n*_ contains no mixed-coordinate derivatives.

In vMCG, this projected nuclear wavefunction is expanded in a time-dependent Gaussian basis,5
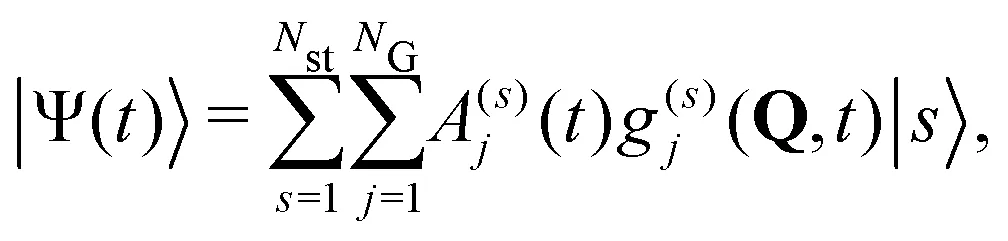
where |*s*〉 is an electronic basis state, *N*_st_ is the number of electronic states, *N*_G_ is the number of Gaussian wavepackets (GWPs), and *A*^(*s*)^_*j*_(*t*) are the expansion coefficients. The notation *g*^(*s*)^_*j*_ allows for the general multi-set form, in which each electronic state has a separate GWP basis. In the single-set formulation used here, all electronic states share the same GWP basis, so *g*^(*s*)^_*j*_(***Q***, *t*) ≡ *g*_*j*_(***Q***,*t*) and the electronic-state label on the GWP is omitted below. State dependence, including population transfer, is encoded in the coupled evolution of the coefficients *A*^(*s*)^_*j*_(*t*). Consequently, only *N*_G_ distinct GWP-centre geometries are required for electronic-structure evaluation at a given time, rather than a separate set for every electronic state.

Each multidimensional GWP is written in quadratic form,

6 *g*_*j*_(***Q***,*t*) = exp(***Q***^T^***ζ***_*j*_***Q*** + ***ξ***_*j*_(*t*)^T^***Q*** + ***η***_*j*_(*t*)).

Here ***ζ***_*j*_ is the width matrix, ***ξ***_*j*_(*t*) encodes the phase-space centre, and ***η***_*j*_(*t*) is a complex phase/normalisation parameter. We use frozen widths, so ***ζ***_*j*_ is time independent and only ***ξ***_*j*_(*t*) and ***η***_*j*_(*t*) are propagated. For diagonal widths the GWP factorises into one-dimensional Gaussians,7
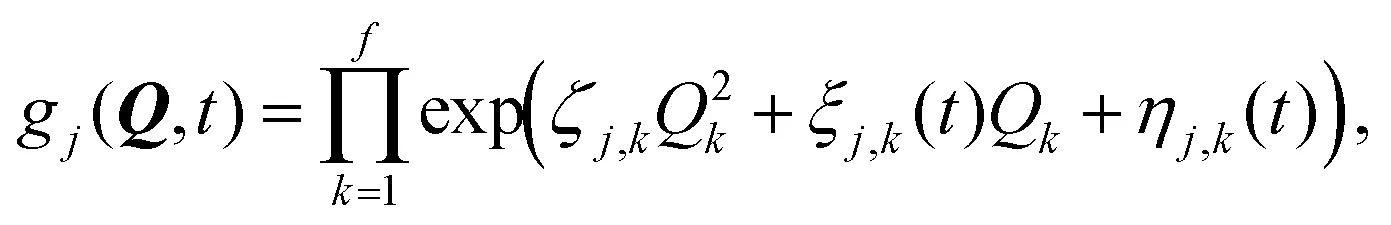
where *f* is the number of nuclear degrees of freedom.

Applying the Dirac–Frenkel variational principle,^42^8
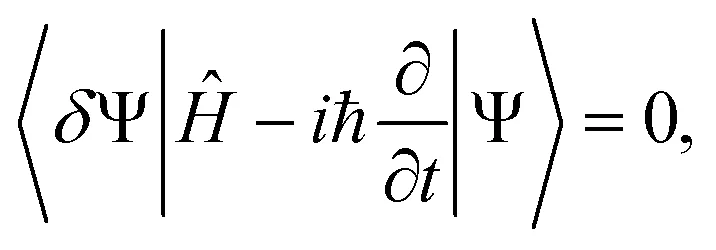


to eqn (5) gives coupled equations of motion for the expansion coefficients and the Gaussian parameters. In compact atomic-unit form,

9 *i***Ȧ** = **S**^−1^(**H** − *iτ*)**A**,

10 *i***Λ̇** = **C**_G_^−1^***Y***,

where **S** is the GWP overlap matrix, **H** is the Hamiltonian matrix, and *τ* is the differential-overlap matrix. The vector **Λ** collects the propagated Gaussian parameters, while **C**_G_ and ***Y*** are the metric and force-like quantities obtained by projecting the TDSE onto the Gaussian parameter space. The subscript on **C**_G_ distinguishes this vMCG parameter-space metric from the adiabatic-to-diabatic transformation matrix introduced below.

vMCG is therefore a variational quantum-wavepacket method rather than an ensemble of independent classical trajectories.^43^ Within the selected electronic subspace, it converges to the projected molecular TDSE only in the limit of a complete Gaussian basis and exact Hamiltonian matrix elements. Practical DD-vMCG calculations introduce additional approximations because the electronic Hamiltonian is evaluated at a finite set of database geometries, local harmonic expansions are used to obtain analytic Gaussian matrix elements, and nearby electronic-structure information is reused by interpolation.

### 2.2 Direct dynamics

In DD-vMCG, the Hamiltonian matrix elements required in eqn (9) and (10) are obtained from electronic-structure calculations performed on the fly. Around a database geometry ***Q***_*j*_, each quasi-diabatic potential-matrix element is represented by the local harmonic expansion11
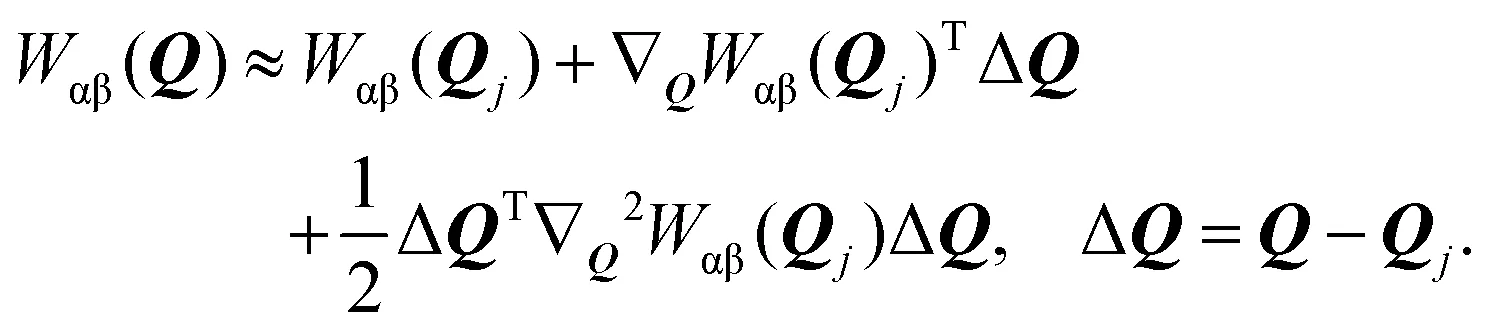


Here ∇_***Q***_*W*_αβ_ and ∇_***Q***_^2^*W*_αβ_ are the nuclear gradient and Hessian of matrix element *W*_αβ_, respectively. This local harmonic approximation allows the corresponding Gaussian matrix elements to be evaluated analytically. At geometries without explicit Hessian calculations, the quasi-diabatic Hessians are obtained by updating previously available Hessians using gradient information.^17,41,44^ Modified Shepard interpolation combines the local quadratic models about nearby database geometries to obtain potential and derivative information at a requested geometry.^17,21,45^ A new electronic-structure calculation is performed when the database-reuse criterion is not satisfied. Further details of Hessian updating and interpolation are given in SI Section S11.1.

### 2.3 Predictor-corrected propagation diabatisation

The local harmonic approximation and modified Shepard interpolation require a consistent quasi-diabatic representation across neighbouring database geometries. Independently calculated electronic eigenvectors have arbitrary phases: for real electronic functions, reversing the sign of one state reverses its pairwise nonadiabatic coupling vectors. Near degeneracies, rapid changes in the adiabatic states character introduce an additional difficulty for continuing the transformation between geometries.^1,8^

We use a predictor–corrector procedure based on the propagation-diabatisation framework.^22,41^ The diabatic gauge is fixed by taking the adiabatic and quasi-diabatic states to coincide at the Franck–Condon geometry, **C**(***Q***_FC_) = **I**. At a new geometry, a local Taylor expansion or Shepard interpolation of the existing quasi-diabatic data provides a predicted potential matrix. Diagonalising this model and transforming its derivatives into the adiabatic representation gives predicted energies, gradients and nonadiabatic coupling vectors for comparison with the electronic-structure results.

#### Regular continuation

When the predicted ordering of the diagonal quasi-diabatic potential-matrix elements is unchanged from that at the selected database reference and no adjacent calculated adiabatic energy gap falls below 10^−8^ Hartree, the ADT matrix is propagated with eqn (12). Before propagation, the sign of each calculated nonadiabatic coupling vector is obtained pairwise against its predicted counterpart. If their dot product is negative, indicating opposite orientation, the calculated vector is multiplied by −1 for that state pair.

The phase-adjusted couplings enter the ADT equation^46^

12 ∇_***Q***_**C**(***Q***) = −**F̲**(***Q***)**C**(***Q***),

where **C**(***Q***) is the adiabatic-to-diabatic transformation (ADT) matrix and **F̲**(***Q***) is the non-adiabatic coupling matrix, with elements13
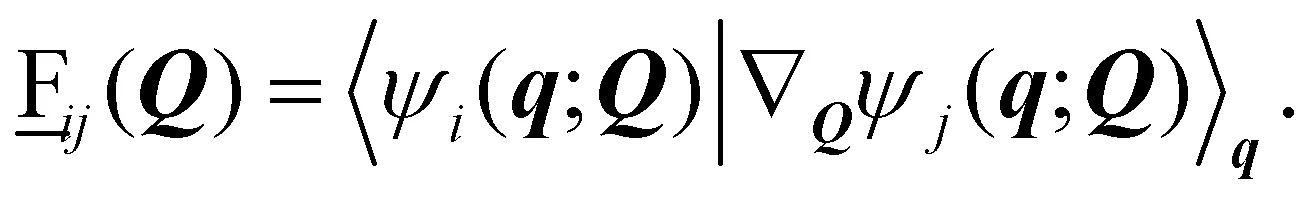


The subscript ***q*** indicates integration over the electronic coordinates. For a short nuclear displacement from ***Q*** to ***Q*** + Δ***Q***, eqn (12) gives the formal path-ordered update14
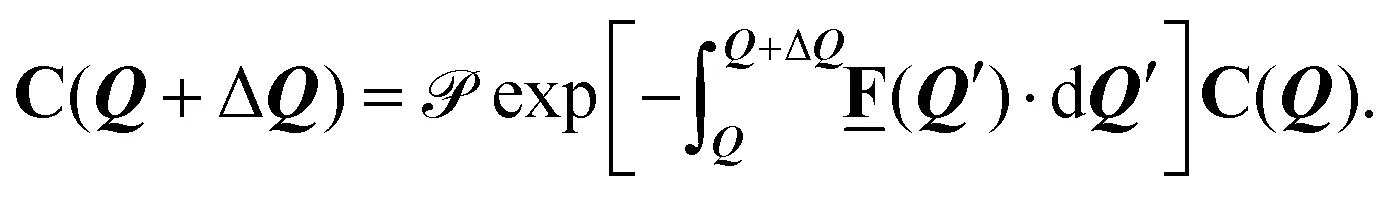


The implementation constructs the couplings along the straight reference-to-geometry segment and evaluates the ADT at the new geometry, ***Q*** + Δ***Q***, using a symmetric propagator.^22,41^

#### Crossing response

The alternative construction of the ADT is activated when the rank ordering of the diagonal quasi-diabatic potential-matrix elements changes between the reference and predicted new geometry, or when adjacent calculated adiabatic energies differ by less than 10^−8^ Hartree. The latter criterion provides a numerical near-degeneracy safeguard, motivated by the non-uniqueness of individual adiabatic states at exact degeneracy. Instead of directly propagating the ADT across this segment, a local cubic quasi-diabatic potential-matrix model is constructed along it.

The constant, linear and quadratic coefficients are fixed by the reference quasi-diabatic potential matrix and its path-projected gradients and Hessians. Only the cubic coefficients are optimised. Each trial model is diagonalised, and its endpoint adiabatic energies and path-projected derivative matrix are compared with the calculated targets at *Q* + Δ*Q*. These comprise the adiabatic energies, projected energy gradients and projected interstate-coupling vectors, **D**_*ij*_ = (*V*_*j*_ − *V*_*i*_)**F̲**_*ij*_. Diagonalisation of the optimised model supplies the ADT matrix. After either branch, the resulting ADT is used to transform the calculated electronic-structure data into the quasi-diabatic representation. The local model and both target contributions are given in SI Section S11.2.

#### Relation to the previous algorithm

The previous algorithm would select a predicted transformation or potential matrix directly following a state-ordering change, a small predicted–calculated coupling overlap or a small adiabatic energy gap.^41^ The present crossing response instead uses the ADT obtained by diagonalising the optimised local cubic quasi-diabatic potential matrix, rather than the ADT from the initial database prediction. A poor coupling overlap is retained as an intruder-like diagnostic but does not independently activate this correction.

These numerical procedures do not remove the formal limitations of propagation diabatisation in a finite electronic subspace. When the retained states do not satisfy the ADT integrability conditions, path dependence and residual derivative couplings may remain.^1,8,22,40,41,47^ Informed sampling addresses the separate dependence on database construction by supplying reference geometries before the wavepacket generates further entries.

## 3 Computational methodology

Electronic-structure calculations were performed with Molpro (version 2022),^35,48^ and the DD-vMCG propagations were carried out with a development version of Quantics.^36,49^ Adiabatic potential surfaces are energy ordered and labelled *S*_0_,*S*_1_,*S*_2_…, with *S*_0_ the lowest energy state. Diabatic states are labelled *X̃*, *Ã*, *B̃*,…, which are identical with *S*_0_, *S*_1_, *S*_2_,… at the FC point. No symmetry constraints were imposed on either the electronic-structure calculations (nosym and noextra) or the dynamics.

### 3.1 Electronic structure calculations

O–O elongation in photoexcited CH_2_OO accesses regions in which several low-lying singlet states become nearly degenerate. A single-reference method may not provide a balanced description of the interacting states in these regions, making a state-averaged multireference treatment appropriate.^4–6^ Because DD-vMCG requires thousands of on-the-fly evaluations of energies, gradients and nonadiabatic coupling vectors, the production level must balance the representation of these multistate interactions against computational cost.

Production DD-vMCG calculations therefore used equal-weight SA-CASSCF as implemented in Molpro.^35,37,48^ The state average comprised the ground state and the seven lowest singlet excited states, defining an eight-state manifold *S*_0_–*S*_7_. The eight-state manifold accounts for the five singlet states correlating with CH_2_O(X^1^A_1_) + O(^1^D) and the three singlet-coupled states correlating with CH_2_O(a^3^A″) + O(^3^P). This follows from the fivefold and threefold orbital degeneracies of the oxygen terms, respectively, combined with spatially nondegenerate formaldehyde states.^33^ Adiabatic energies, nuclear gradients and nonadiabatic coupling vectors (Hessians only at the FC) were evaluated at this level. The omission of dynamic correlation can affect the quantitative excitation energies and dissociation energetics and may consequently affect predicted channel populations.

An active space of 10 electrons in 8 orbitals, denoted CASSCF(10e,8o), was used with the 6-31G(d,p) basis set for all on-the-fly calculations. The active space was chosen to include the principal valence orbitals involved in C–O and O–O bonding, low-lying valence excitations and O–O bond cleavage: σ(CO), σ*(CO), π(CO), π*(CO), σ(OO), σ*(OO), and two p-type nonbonding orbitals localised predominantly on the terminal oxygen. The active orbitals are shown in Fig. 1.

**Fig. 1:**
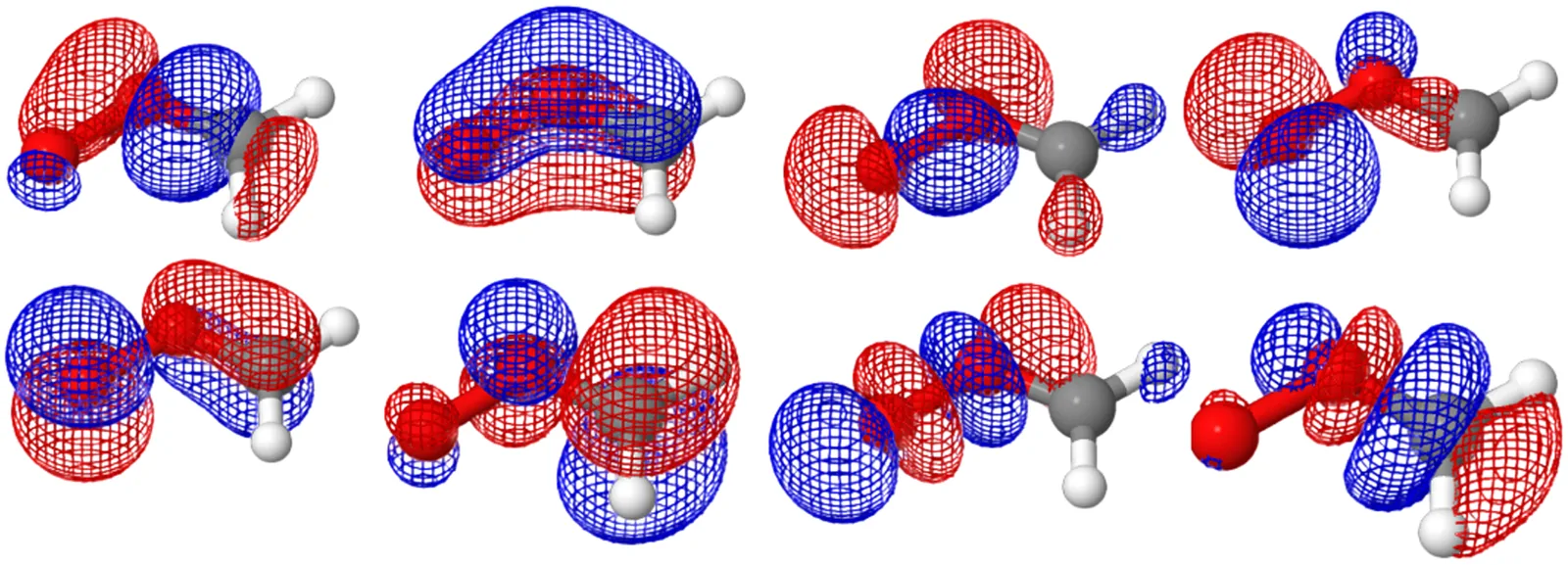
Isosurfaces of the eight molecular orbitals included in the CASSCF(10e,8o) active space for CH_2_OO. Red and blue meshes denote opposite orbital phases.

To examine how the production level represents the state ordering, near-degeneracy pattern and channel separation along the selected dissociation coordinate, we calculated one-dimensional scans as a function of O–O distance (Fig. 2). SA-CASSCF was compared with complete active space second-order perturbation theory (CASPT2) and extended multistate CASPT2 (XMS-CASPT2) using CAS(10e,8o) and CAS(12e,10o) reference spaces.^38,50^ Both perturbative treatments used a real level shift of 0.3 a.u. to reduce sensitivity to intruder states and an ionisation-potential–electron-affinity (IPEA) shift of 0.25 a.u. These scans serve only as coordinate-specific electronic-structure diagnostics; all DD-vMCG calculations reported below used SA-CASSCF(10e,8o)/6-31G(d,p). Dawes *et al.* reported upper-limit estimates of ≤2.12 eV (49.0 ± 0.3 kcal mol^−1^) for O(^1^D) + H_2_ CO(X^1^*A*_1_) and ≤3.28 eV (75.7 ± 0.3 kcal mol^−1^) for O(^3^ P) + H_2_ CO(a^3^*A*^''^), giving a separation of 1.16 eV between the quoted limits.^26^

**Fig. 2:**
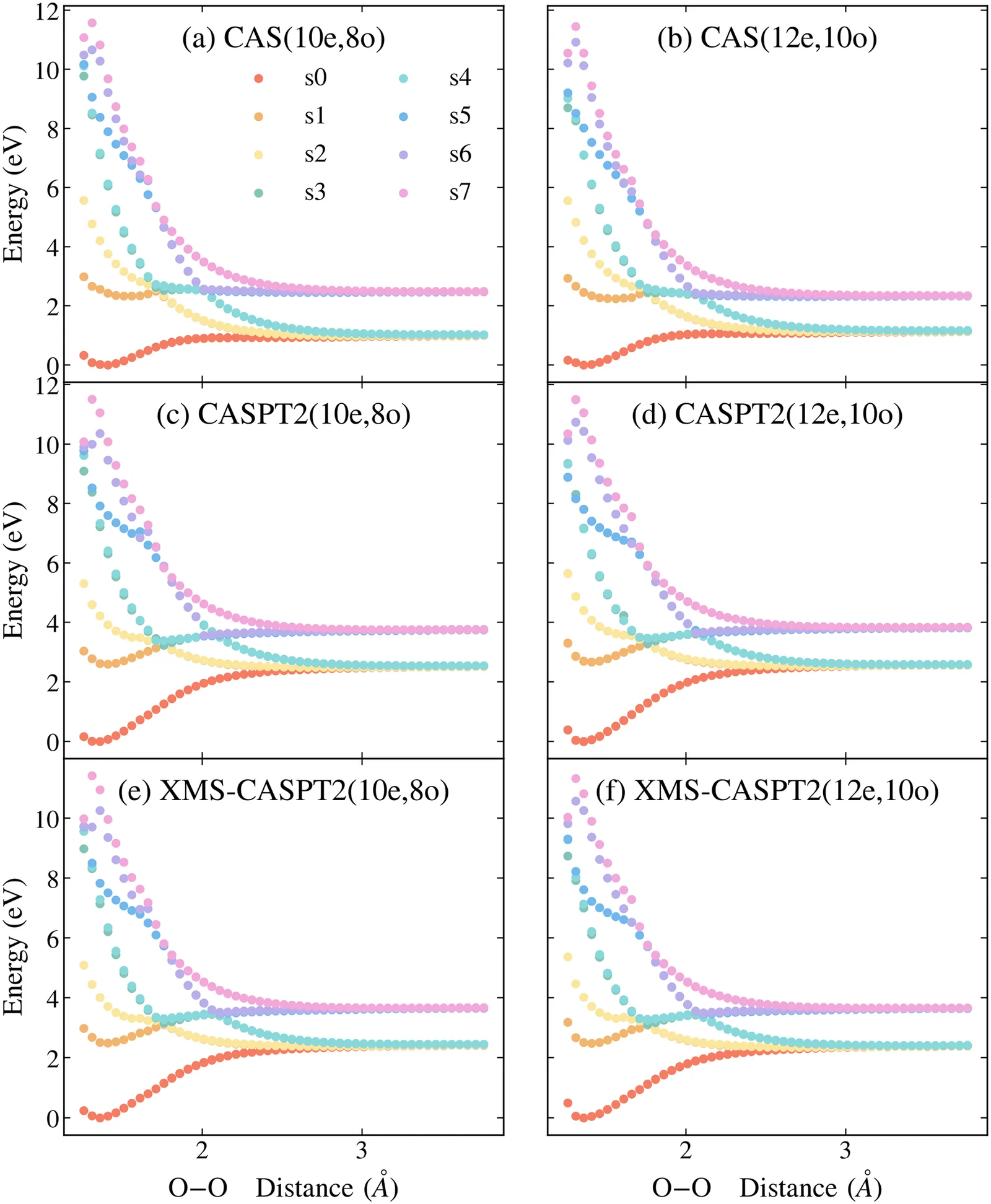
Potential-energy profiles of the eight lowest singlet states of CH₂OO along the one-dimensional O–O dissociation scan. Panels (a, b), (c, d) and (e, f) show SA-CASSCF, CASPT2 and XMS-CASPT2, respectively. The left-hand panels (a, c, e) use CAS(10e,8o), and the right-hand panels (b, d, f) use CAS(12e,10o) reference spaces.

Previous studies have examined both the dissociation dynamics and the spectroscopic performance of CASSCF-based models of CH_2_OO. Esposito *et al.* conducted full-dimensional surface hopping at SA7-CASSCF(10e,8o)/aug-cc-pVDZ.^27^ Antwi *et al.* used a CASSCF(10e,8o) reference for SS-SR-CASPT2/cc-pVDZ dynamics, propagating 199 trajectories over *S*_0_–*S*_6_ and examining the role of long-range internal conversion in product-state formation.^28^ In complementary nuclear-ensemble absorption calculations at CASPT2/aug-cc-pVDZ, McCoy *et al.* found that enlarging the active space to (12e,10o) red-shifted the spectrum and improved agreement with experiment.^51^ These results motivate testing active-space and correlation effects in the O–O cuts of Fig. 2. These examples reflect the studies that initially informed our model selection, within a wider body of quantum-dynamics and trajectory work on CH_2_OO.^30,31,33,52^ Broader accounts are available in the reviews by Karsili *et al.* and Nikoobakht *et al*.^29,53^

Here the comparison tests whether the working model retains the multistate interaction pattern needed to examine informed sampling, rather than whether it reproduces the absorption maximum. Along this cut, perturbative correlation changes the approach to dissociation and the channel energies, while preserving the two asymptotic state groups and their degeneracies. For CAS(10e,8o), the upper–lower group separation is 1.49 eV at SA-CASSCF, 1.22 eV at CASPT2 and 1.23 eV at XMS-CASPT2; the corresponding CAS(12e,10o) values are 1.22, 1.27 and 1.27 eV. The perturbative treatments also move the dissociation limits towards the reported upper-limit estimates. These scans support using SA-CASSCF(10e,8o)/6-31G(d,p) to test the sampling and diabatisation workflow in eight states and all nine vibrational coordinates at tractable cost. They do not establish full-dimensional quantitative accuracy.

### 3.2 Coordinate system and initial wavepacket

CH_2_OO is a non-linear five-atom system with *f* = 3*N* − 6 = 9 vibrational degrees of freedom. Nuclear motion was represented in rectilinear mass- and frequency-weighted normal coordinates, ***Q*** = (*Q*_1_,…,*Q*_*f*_), obtained from a coupled-cluster singles-and-doubles (CCSD)/6-311++G(d,p) harmonic frequency calculation at the ground-state minimum. This coordinate transformation was held fixed throughout the dynamics. The coordinate *Q*_3_ is dominated by O–O stretching, with negative displacement corresponding to O–O elongation; all normal-coordinate displacements reported below are in dimensionless coordinates.

The initial harmonic vibrational ground-state wavefunction was represented using an *N*_G_ = 32 frozen GWP basis with a common width parameter of 
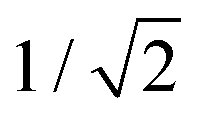
 (coordinate standard deviation) for each mode.

All GWPs were initially centred at the FC geometry and distributed in momentum space. Only the zero-momentum GWP had a non-zero initial coefficient; the remaining GWPs supplied variational flexibility during propagation. The electronic initial condition was a vertical promotion to the optically bright *S*_2_ state, consistent with the assignment of the near-UV band to a π* ← π excitation localised on the carbonyl oxide moiety.^23–26^ Because the adiabatic and quasi-diabatic bases coincide at the FC geometry under the chosen gauge, this initial condition corresponds to population of *B̃* in the propagated quasi-diabatic basis.

### 3.3 DD-vMCG propagation

The nuclear wavefunction was propagated with the DD-vMCG implementation in Quantics.^36^ Electronic-structure quantities were evaluated on the fly at geometries requested by the evolving GWP basis and stored in an electronic-structure database. Around each database geometry, a local harmonic approximation (LHA) to the quasi-diabatic Hamiltonian was constructed from the stored energies, gradients and Hessians. When a requested geometry satisfied the database-reuse criterion, the Hamiltonian information was obtained by modified Shepard interpolation; otherwise, a new electronic-structure calculation was performed and the resulting point was added to the database.^17,21,45^

The threshold for generating a new electronic-structure point was dbmin = 0.1 Bohr. In Quantics, this quantity is defined as the maximum Cartesian displacement of any atom relative to the nearest database geometry. Hessians of the eight adiabatic potential energies were calculated explicitly only at the FC reference geometry. At the remaining seed geometries and geometries added during propagation, the electronic-structure calculations supplied adiabatic energies, their gradients and nonadiabatic coupling vectors. These data were transformed using the ADT and incorporated into the quasi-diabatic Hessian-updating and interpolation procedure described above in Sections 2.2 and 2.3.

To simulate dissociation and avoid numerical difficulties at very long O–O distances of the represented dissociation region, a complex absorbing potential (CAP) was applied along *Q*_3_.^54^ The Hamiltonian was augmented as *Ĥ* → *Ĥ* − *iW*(*Q*_3_), with

15 *W*(*Q*_3_) = *η*Θ[*k*(*Q*_3_ − *Q*^CAP^_3_)]^*n*^,

where *η* is the CAP strength, Θ is the Heaviside step function, *n* is the polynomial order, and *k* = ±1 selects the absorbing direction. We used *k* = −1, so the absorber acts for *Q*_3_ < *Q*^CAP^_3_. Its onset was *Q*^CAP^_3_ = −25, corresponding to approximately *R*_OO_ = 3.1 Å along the reference cut, with *η* = 0.005 a.u. and *n* = 3. In the DD-vMCG implementation used here, GWPs crossing the CAP dividing surface are uncoupled from the remaining active basis, thereby preventing their subsequent interaction with the unabsorbed wavepacket.

For the present comparison, each database was built through repeated 50 fs read–write propagations. During each propagation, electronic-structure calculations at newly requested geometries supplied additional database entries, while nearby existing information was reused by interpolation. The accumulated database was retained and rediabatised between propagations, proceeding outwards from the FC reference. This procedure was repeated until each database contained approximately 5 × 10^3^ points. Observables and wavefunctions from these growth runs were recorded at 0.5 fs intervals.

The population and nuclear-motion results reported below were obtained from database-reuse (read-only) propagations, so the Hamiltonian was evaluated exclusively from the prebuilt database without additional quantum-chemistry calculations. For these runs, the properties and wavefunction output interval was 0.1 fs, providing a fine temporal sampling of the analysed observables. Additional read–write propagations were analysed for energy drift, adaptive RK5 effort and electronic-energy prediction errors, with their settings documented separately in SI Section S9.

### 3.4 Informed sampling protocol

In the conventional dark-start workflow examined here, no dissociation-path geometries were inserted before exploratory propagation from the FC initial condition. That propagation generated the first database entries, including energies, gradients, nonadiabatic coupling vectors and ADT data; the resulting database was then rediabatised before reuse.

To reduce this initialisation sensitivity, we introduced an informed-sampling protocol. Before starting the database-growth propagations, a set of reference geometries was generated along *Q*_3_ at intervals of Δ*Q*_3_ = 0.25 a.u., spanning *Q*_3_ = −20.00 to 4.00 a.u. and comprising 97 geometries. Electronic-structure continuation was performed in two scans initiated from a geometry with *R*_OO_ = 1.77 Å. One scan proceeded towards shorter O–O distances and the FC region, while the other proceeded towards increasing O–O elongation. Initiating both scans from this interior geometry limits dependence of the CASSCF orbital continuation on either endpoint of the sampled coordinate range.

At each pre-sampled geometry, the eight-state SA-CASSCF(10e,8o)/6-31G(d,p) quantities required by DD-vMCG were calculated. The orbital guess at each step was taken from the nearest previously converged geometry in the same scan to promote continuity of the CASSCF solutions. The 97 additional seed entries and the common FC entry formed a 98-entry initial database, which was rediabatised using the two-branch predictor-corrector procedure described in Section 2.3. These entries provide reference coverage along the dissociation coordinate before the 9D wavepacket adds further database points.

The 97 additional seed calculations required 11.2 h of summed elapsed time (recorded in the output), excluding the FC calculation common to both workflows. The subsequent database-growth runs required 3955 electronic-structure calls for informed sampling and 4094 for dark-start, a reduction of 3.40%. Including the additional seed and shared FC calculations gives 4053 and 4095 calls, respectively, *i.e.* no significant change in the overall number of calculations. Stage-resolved seed timings and growth-call counts are reported in SI Section S11.3.

For the comparison in Section 4, the dark-start and informed-sampling databases were each grown to approximately 5 × 10^3^ points under matched propagation settings. They are not claimed to be converged global representations of the 9D quasi-diabatic Hamiltonian. Instead, they provide controlled test cases for assessing how database initialisation affects Hamiltonian smoothness, electronic-state correlation and the interpretation of wavepacket observables.

## 4 Result and discussion

### 4.1 Quasi-diabatic Hamiltonian quality along the dissociation coordinate

We first assess the quasi-diabatic Hamiltonian along the coordinate most directly associated with O–O elongation. Fig. 3 evaluates the same interpolated LHA used during propagation along a common one-dimensional path in *Q*_3_. This cut is neither a global representation of the 9D Hamiltonian nor a direct measure of its off-diagonal couplings. It instead tests whether the diagonal elements vary smoothly and retain a coherent crossing pattern along the selected dissociation coordinate. The visual comparison is supplemented by a coordinate-specific curvature diagnostic: the matched production-database comparison is retained in the main text, while the state-resolved initial-database comparison is provided in the SI.

**Fig. 3:**
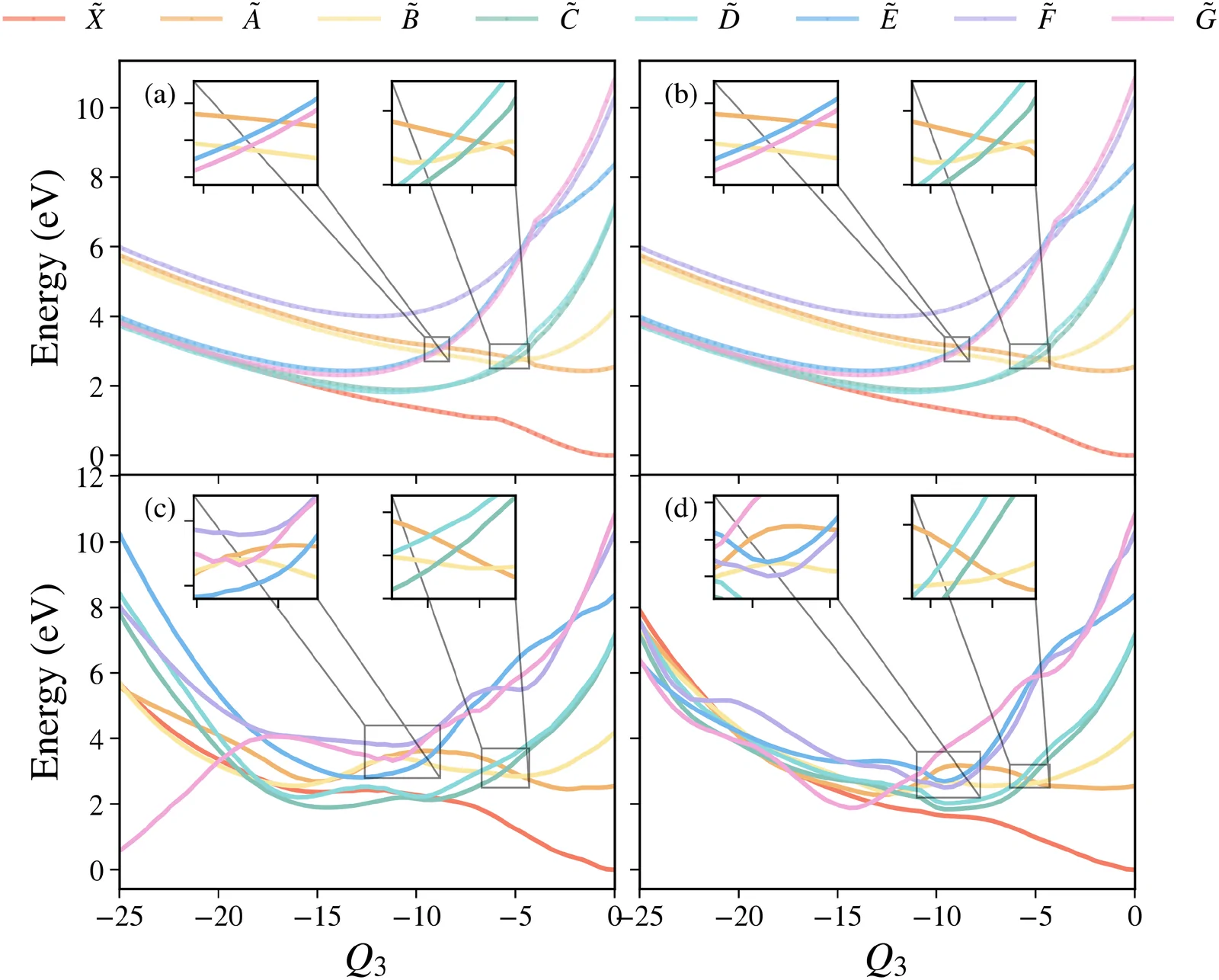
One-dimensional cuts of the diagonal quasi-diabatic potential-matrix elements *W*_αα_(*Q*_3_) along the O–O-stretch-dominated coordinate *Q*_3_. The same Shepard-interpolated LHA used during propagation is evaluated on a common grid, with all other normal coordinates fixed at their FC values. In each panel, the energies are shifted so that the ground-state energy at the FC geometry is zero. Panels show (a) the rediabatised initial informed-sampling database (the FC entry and 97 additional seed entries), (b) the informed-sampling production database (∼5 × 10^3^ points, *N*_G_ = 32), (c) the dark-start initial database (*N*_G_ = 32), and (d) the dark-start production database (∼5 × 10^3^ points, *N*_G_ = 32). Insets enlarge the boxed multistate interaction regions. CoIn1 lies closer to the FC geometry, whereas CoIn2 occurs at more negative *Q*_3_.

#### Initial quasi-diabatic potentials

Here, initial denotes the earliest quasi-diabatic potentials available for each workflow: the rediabatised surfaces before production dynamics for informed-sampling, and the database obtained after the first DD-vMCG run for dark-start. Before substantial database growth, the initial informed-sampling database (Fig. 3a) gives smooth diagonal elements across the diagnostic range and resolves both interaction windows as compact sequences of crossings between quasi-diabatic states. Following the cut from the FC region towards decreasing *Q*_3_, CoIn1 is encountered first. Within this window, the *Ã* and *B̃* states first form a clear X-shaped crossing, which reverses their local energy order. Immediately beyond this crossing, *Ã* lies above *B̃*; the near-parallel *C̃*/*D̃* states subsequently descend through the reordered pair and therefore intersect *Ã* before *B̃*. CoIn1 is consequently better described as an ordered cluster of pairwise crossings than as a single simultaneous four-state crossing. By CoIn2, *Ã* and *B̃* retain their post-CoIn1 ordering and run nearly parallel through the interaction region rather than crossing one another again. The *Ẽ*/*G̃* states descend through this pair, again intersecting *Ã* before *B̃*. Thus, the two windows involve related state subsets but differ in their local crossing structure. In both cases the participating diagonal elements cross without developing pronounced avoided-crossing-like repulsion, and the *W*_αα_ curves vary smoothly outside the interaction regions. This ordered sequence provides the reference for interpreting the adiabatic-character relabelling discussed below, but the diagonal curves alone do not determine interstate coupling strengths or population-transfer probabilities.

The *N*_G_ = 32 dark-start initial Hamiltonian (Fig. 3c) retains a recognisable CoIn1 crossing pattern, but differs more substantially within CoIn2. Rather than the compact *Ã*/*B̃*–*Ẽ*/*G̃* exchange obtained from the initial informed-sampling database, the dark-start cut shows additional closely spaced intersections and local bending involving *Ã*/*B̃* and the upper *Ẽ*/*F̃*/*G̃* states. These features establish that the diagonal cut is less regular in the congested upper-state region. They are consistent with residual sampling-history dependence in the interpolated quasi-diabatic representation, although the diagonal elements alone cannot separate gauge irregularity from interpolation error.

#### Curvature-based smoothness metric

To compare the bending of the diagonal quasi-diabatic cuts on a common grid, we define the displacement from the FC geometry along the dissociative direction as

16 *s* = *Q*^FC^_3_ − *Q*_3_, *s* ≥ 0, *Q*^FC^_3_ ≈ 0,

and express each diagonal cut as

17 *E*_α_(*s*) = *W*_αα_(*Q*^FC^_3_ − *s*).

The second derivative of *E*_α_ measures its local bending. Squaring this derivative prevents positive and negative curvatures from cancelling, and integrating accumulates their contributions along the cut:18
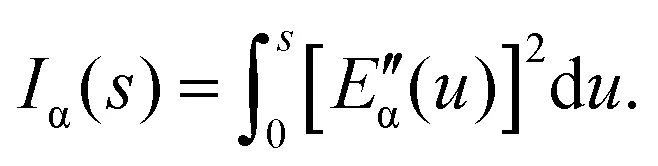


Dividing by the sampled interval and taking the square root gives the running root-mean-square curvature for that state:19
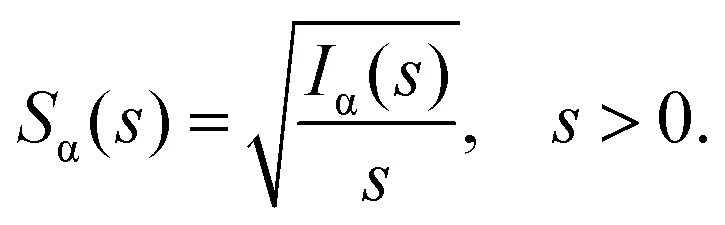


We sum these values over the retained quasi-diabatic states to obtain the aggregate diagnostic,20
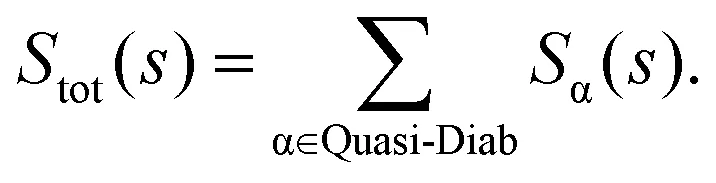


We report *S*_tot_(*s*_max_) at the end of the scan. This quantity is coordinate dependent, is sensitive to numerical differentiation and is not a molecular observable. It is used only to compare cuts evaluated on the same coordinate grid with the same interpolation and differentiation procedures.

The state-resolved initial-database curves are reported in SI Fig. S8. The initial informed-sampling database gives *S*_tot_ (*s*_max_) ≈ 0.885, compared with 1.26 for the *N*_G_ = 32 dark-start initialisation and 3.06 for the deliberately sparse *N*_G_ = 4 dark-start initialisation. The matched *N*_G_ = 32 comparison therefore corresponds to an approximately 30% lower aggregate curvature for the initial informed-sampling database. The *N*_G_ = 4 result is included as a sensitivity test rather than as a matched estimate of the protocol effect. In the dark-start calculations, the largest additional curvature contributions arise from the upper quasi-diabatic states and extend across a broader interval containing CoIn2.

#### Quasi-diabatic Hamiltonians after database growth

We next compare cuts extracted from production databases of similar size (Fig. 3b and d). In the informed-sampling workflow, the production profile remains close to the initial informed-sampling profile. CoIn1 retains the initial *Ã*/*B̃* X-shaped crossing followed by passage of *C̃*/*D̃*, and CoIn2 retains the near-parallel *Ã*/*B̃* pair crossed by *Ẽ*/*G̃*. The diagonal elements also remain smooth outside these interaction windows. The persistence of this ordered structure after growth to approximately 5 × 10^3^ points is consistent with the pre-sampled geometries continuing to provide a stable reference for the propagated quasi-diabatic representation.

In the dark-start production database, CoIn1 remains recognisable, but growth does not restore the ordered CoIn2 pattern obtained with informed-sampling (Fig. 3d). Instead, CoIn2 contains a broader cluster of closely spaced intersections and pronounced local bending involving *Ã*/*B̃* and the upper *Ẽ*/*F̃*/*G̃* states. The pairwise sequence resolved in the informed-sampling cut cannot therefore be assigned unambiguously from the dark-start diagonal elements. Relative to the informed-sampling cut, the upper-state region remains less regular after comparable database growth. This observation motivates the explicit state-character analysis in the following subsection; the diagonal potentials alone do not establish a unique asymptotic state correlation.

The production-database curvature profiles support the visual comparison (Fig. 4). The informed-sampling database gives *S*_tot_ (*s*_max_) ≈ 0.851, close to its initial-database value of 0.885, whereas the dark-start production database gives 1.64. Thus, on this common diagnostic grid, the informed-sampling value is approximately 48% lower. The dark-start value also exceeds its *N*_G_ = 32 initial value of 1.26, so the data do not support a claim that database growth alone regularises the diagonal Hamiltonian. The state-resolved profiles instead show continued curvature accumulation in the highest retained states, particularly *F̃* and *G̃*, over a wider portion of the scan. Within this comparison, subsequent database growth therefore does not bring the *dark-start* cut to the smoothness obtained from informed-sampling.

**Fig. 4:**
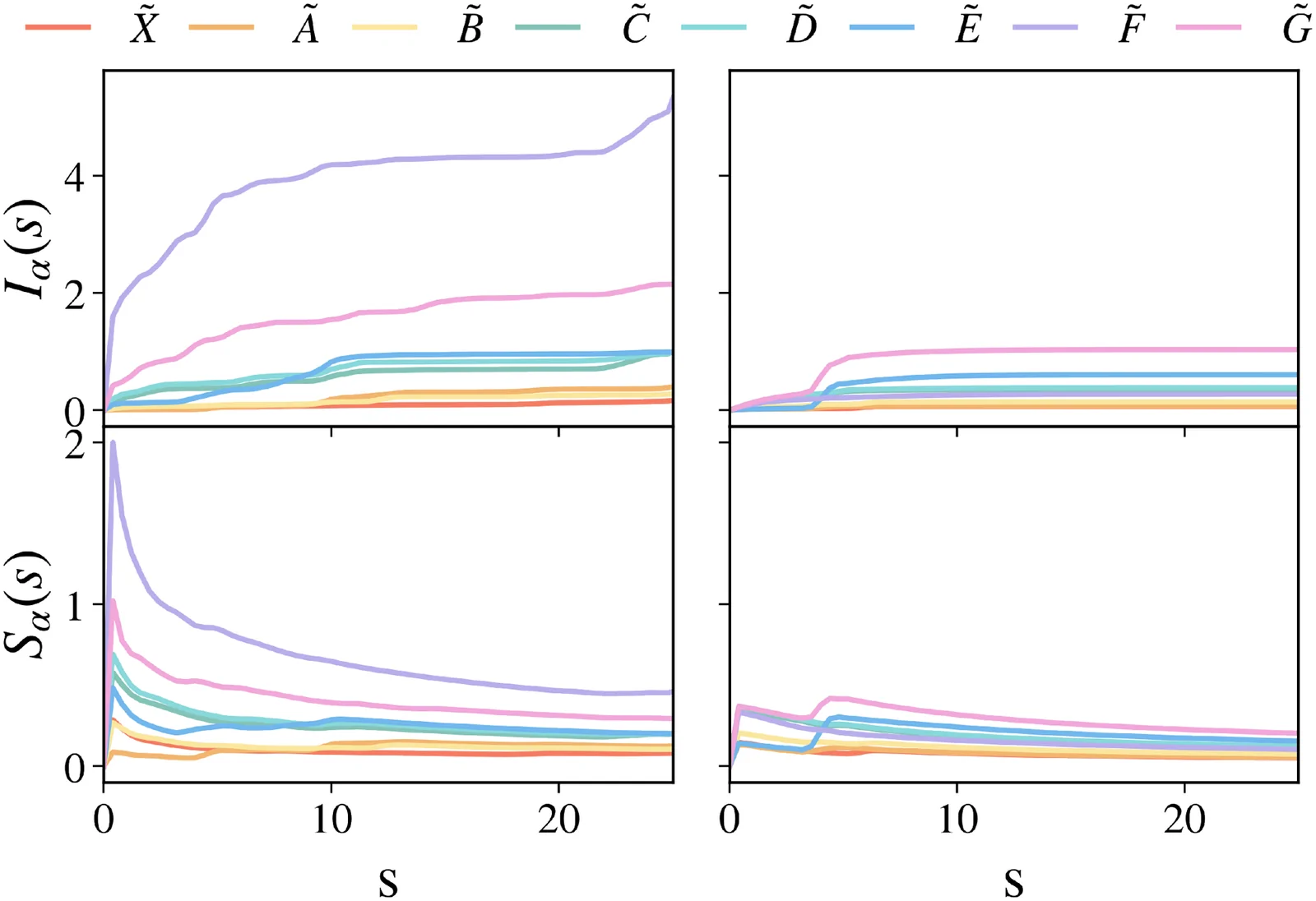
State-resolved accumulated squared curvature *I*_*α*_(*s*) (top) and running RMS curvature *S*_α_(*s*) (bottom) for the one-dimensional cuts *W*_αα_(*Q*_3_) obtained from the production databases. Left: Dark-start workflow. Right: Informed-sampling workflow. Both databases contain approximately 5 × 10^3^ points and were generated with *N*_G_ = 32. The coordinate *s* = *Q*^FC^_3_ − *Q*_3_ measures displacement from the FC region towards O–O elongation. Each coloured curve denotes one quasi-diabatic state.

#### Sensitivity to database initialisation

The broader set of initial cuts in the SI shows that dark-start databases generated with different initial GWP bases vary in both smoothness and crossing structure through the congested region (SI Fig. S1(a–d)). The corresponding informed-sampling cuts vary less across the tested basis sizes (SI Fig. S2(a–d)), because each calculation begins from the same initial database containing the FC entry and 97 additional dissociation-path seed entries. This comparison demonstrates reduced initialisation sensitivity for the tested choices; it does not by itself establish convergence with respect to GWP number or anchor-set construction.

To examine other directions, we also extracted cuts along *Q*_1_, *Q*_2_, *Q*_5_, *Q*_6_ and *Q*_8_ from the final approximately 5000-point databases, varying each selected coordinate while holding all remaining coordinates fixed (SI Fig. S9 and S10). These comparisons do not establish global 9D convergence or a strictly diabatic electronic basis.

### 4.2 Adiabatic composition along the O–O dissociation coordinate

The diagonal cuts establish the smoothness and crossing structure of the quasi-diabatic Hamiltonian, but do not by themselves show how its states correlate with the energy-ordered adiabatic manifold. We therefore analyse the stored ADT matrices along a reference O–O dissociation path. Unlike the pure-*Q*_3_ scan used for seeding, this path contains simultaneous displacements in *Q*_1_, *Q*_3_, *Q*_5_, *Q*_6_, *Q*_7_, *Q*_8_ and *Q*_9_. It therefore follows a one-dimensional curve through several normal coordinates and extends away from the seed line.

The reference geometries are matched to distinct DD-vMCG database structures through the global minimum-cost one-to-one assignment described in SI Section S2; this constraint minimises the total mapping cost but does not guarantee that every assigned structure is the independent minimum-cost match to its reference geometry under the selected coordinate-space metric. At a mapped geometry ***Q***, the contribution of adiabatic state *i* to quasi-diabatic state *α* is

21 *w*_*i *→ *α*_(***Q***) = |**C**_*iα*_(***Q***)|^2^,

and the dominant adiabatic index is defined as

22 *i*_α_(***Q***) = argmax_*i*_|**C**_*i*α_(***Q***)|^2^

The colour maps in Fig. 5 report *i*_*α*_ for each fixed quasi-diabatic label *α*. A change in colour therefore denotes a change in the dominant adiabatic component of that quasi-diabatic state, rather than a relabelling of the quasi-diabatic basis itself. Because the dominant index discards information about subdominant components, the full composition weights are reported in SI Fig. S3 and S4.

**Fig. 5:**
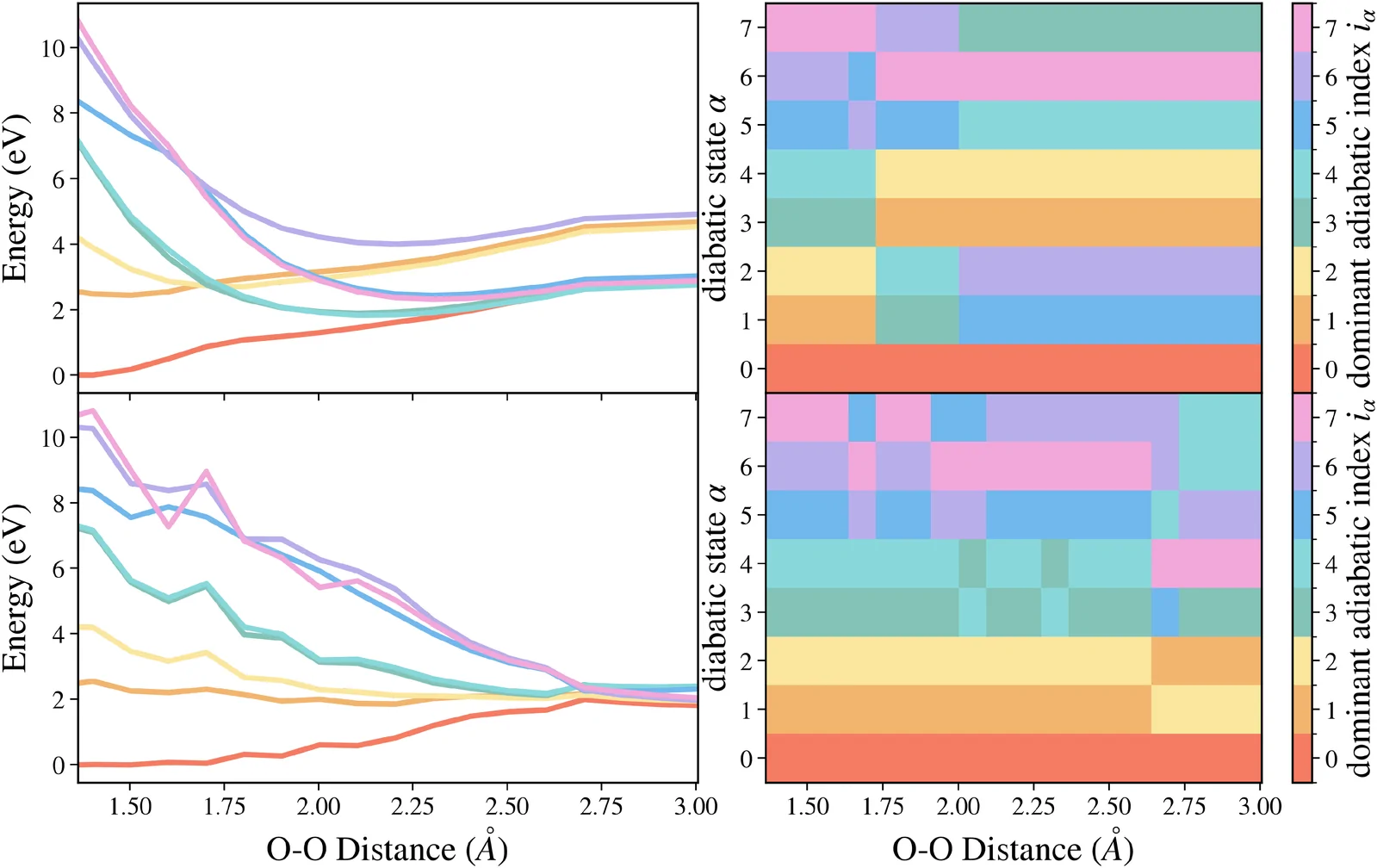
Mapped diagonal quasi-diabatic potential-energy curves and corresponding adiabatic-character maps along an O–O dissociation scan. Left column: diagonal quasi-diabatic potentials *W*_αα_(*R*_OO_). Right column: maps of the dominant adiabatic index, *i*_α_(*R*_OO_) = argmax_*i*_|**C**_*i*α_(*R*_OO_)|^2^, showing the dominant adiabatic component of each quasi-diabatic state *α*. Reference geometries are matched one-to-one to distinct structures in each DD-vMCG database by a global minimum-total-cost assignment, after which the stored ADT matrices are extracted. Top row: informed-sampling workflow. Bottom row: dark-start workflow. CoIn1 lies near *R*_OO_ ≈ 1.7 Å and involves the *S*_1_/*S*_2_ and *S*_3_/*S*_4_ groups; CoIn2 lies near *R*_OO_ ≈ 2.0 Å and involves the *S*_3_/*S*_4_ and *S*_5_/*S*_6_ groups.

For the informed-sampling workflow (Fig. 5, top row), the dominant adiabatic index changes in a structured and localised manner. On the FC side of the scan, *X̃*, *Ã* and *B̃* are dominated by *S*_0_, *S*_1_ and *S*_2_, respectively. At CoIn1, *Ã*/*B̃* exchange dominant character with *C̃*/*D̃*: beyond this window, *Ã*/*B̃* carry the *S*_3_/*S*_4_ character, while *C̃*/*D̃* carry the lower *S*_1_/*S*_2_ character. The same interval contains a higher-state rearrangement involving *Ẽ*, *F̃* and *G̃*. This feature is not a simple pairwise exchange. The *Ẽ* state participates only over a narrow interval before recovering its original dominant index, whereas *F̃* and *G̃* retain exchanged dominant indices on the far side of CoIn1. Its apparent three-state character should be treated cautiously because it occurs at the upper boundary of the retained eight-state manifold, where coupling to excluded states may alter the detailed assignment.

At CoIn2, *Ã*/*B̃* undergo a second exchange, now with *Ẽ*/*G̃*. Beyond this window, *Ã* and *B̃* carry dominant *S*_5_ and *S*_6_ character, respectively, while *Ẽ* and *G̃* carry dominant *S*_4_ and *S*_3_ character. The latter two states become nearly degenerate towards the dissociation limit; their separate dominant-index assignments are therefore less robust than the block-wise conclusion that the *S*_3_/*S*_4_ character has moved into the *Ẽ*/*G̃* subspace. The full weights in the SI likewise indicate increasing mixing within this pair. The significant feature is consequently the collective exchange between state groups, rather than the assignment of every near-degenerate state to a unique adiabatic index.

The mapped diagonal potentials provide a complementary coordinate-space reference for these changes (Fig. 5, top-left). In the informed-sampling workflow, the block-wise changes in dominant adiabatic character are co-localised with the intervals in which the corresponding quasi-diabatic potentials cross. This agreement is important: it distinguishes an interpretable change caused by reordering within a compact interaction region from isolated changes of dominant index distributed across otherwise smooth portions of the scan. It does not, however, quantify the coupling strengths within either window.

The dark-start workflow gives a less coherent correspondence (Fig. 5, bottom row). The *X̃* state retains dominant *S*_0_ character, and *Ã*/*B̃* retain their initial *S*_1_/*S*_2_ assignments until a late exchange at extended O–O distances. By contrast, several mid- and high-lying quasi-diabatic states exhibit short-range changes of dominant index over a broad interval, rather than the two compact block-wise exchanges found with informed-sampling. The mapped dark-start potentials also provide less clearly separated crossing sequences, particularly in the upper manifold. The character changes are therefore not consistently co-localised with a unique set of resolved quasi-diabatic crossings. Together with the initialisation sensitivity established above, this pattern is consistent with a quasi-diabatic representation that remains more dependent on local database history and is consequently less suitable for unique asymptotic state correlation.

### 4.3 Nuclear dynamics

We next compare the wavepacket dynamics generated from the dark-start and informed-sampling databases. The analysis separates two complementary questions: how amplitude is redistributed among the quasi-diabatic states included in the propagation, and how the coupled GWP basis moves along the dissociation-dominated coordinate.

#### Population transfer

In a closed calculation the wavefunction norm is conserved. Here, however, the CAP removes amplitude that reaches *Q*_3_ < *Q*^CAP^_3_, so |Ψ(*t*)|^2^ decreases once the outgoing wavepacket enters the absorbing region. Fig. 6 reports this norm together with the absolute quasi-diabatic state populations. The populations are not renormalised by the instantaneous norm and therefore decrease as amplitude is absorbed.

**Fig. 6:**
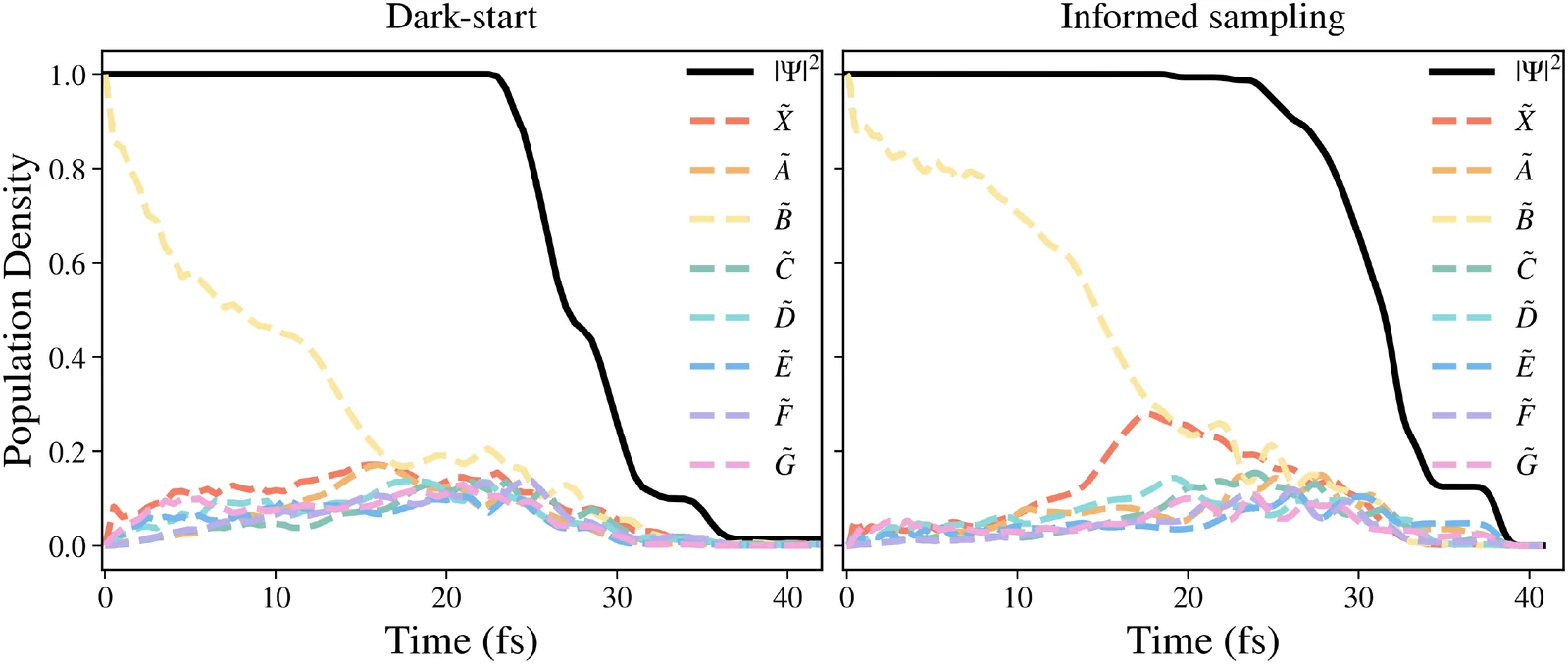
Time evolution of the total wavefunction norm |Ψ(*t*)|^2^ (black solid line) and absolute quasi-diabatic electronic-state populations (coloured dashed lines) during DD-vMCG propagation with a CAP. Left: Dark-start workflow. Right: Informed-sampling workflow. The initial population is placed in *B̃*, which coincides with *S*_2_ at the FC geometry. The populations include the non-orthogonal GWP overlap contributions and are not renormalised by the instantaneous norm; their sum therefore follows the decreasing norm after CAP absorption begins.

Both calculations redistribute population from the initially occupied *B̃* state into several quasi-diabatic states before substantial norm loss. No single receiving state accounts for the full transfer, consistent with the multistate crossing structure identified above. Changes within the first few femtoseconds should not be assigned to passage through a distant interaction window: they also include the immediate evolution of the vertically prepared state under the coupled quasi-diabatic Hamiltonian near the FC region.

The temporal pattern differs between the workflows. In the dark-start calculation (Fig. 6, left), *B̃* begins to decay immediately and its population is distributed gradually across the other seven states; several components reach similar values of approximately 0.1–0.2 before absorption. With informed-sampling (right), *B̃* retains approximately 0.8 population through the first 8 fs and undergoes its largest depletion over approximately 10–18 fs. The principal receiving component is a transient *X̃* population, which reaches approximately 0.3 near 18 fs, while *C̃*, *D̃* and the higher quasi-diabatic states acquire smaller populations. The informed-sampling calculation therefore gives a more temporally concentrated redistribution in the propagation basis. Because quasi-diabatic populations are representation dependent, this comparison does not by itself identify transfer between energy-ordered adiabatic states or product channels; that question is addressed below using the adiabatic projectors.

The norm profiles show when amplitude reaches the absorbing region. The dark-start norm remains close to unity until approximately 24 fs and then falls rapidly, whereas the principal decay in the informed-sampling calculation occurs later and over a broader interval, approximately 25–34 fs. In both calculations, substantial electronic redistribution has begun before the principal norm loss.

#### Motion along the dissociative coordinate and projected phase space

To follow motion along the dissociative coordinate, Fig. 7(a) shows the QUANTICS coordinate expectation 〈*Q*_3_〉 and reported width Δ*Q*_3_. These moments include the finite widths and overlap contributions of the GWPs. Fig. 7(b and c) combines the reported coordinate and momentum expectation values in projected phase-space portraits. The corresponding moment definitions are given in SI Section S1.1. These reduced quantities describe how the finite basis represents the dominant dissociative motion; they are not substitutes for the full 9D nuclear density or channel-resolved flux. The underlying unweighted GWP-centre trajectories are reported in SI Fig. S5 and S6. Both coordinate expectation values initially move towards decreasing *Q*_3_, establishing the same coarse direction of O–O extension. Their early trajectories overlap closely, after which the dark-start mean advances modestly ahead of the informed-sampling mean through much of the principal descent. The shaded intervals overlap substantially, so Fig. 7(a) does not support a general claim that either wavepacket remains more localised throughout this interval. The clearest distinction appears near and after entry into the absorbing region: the informed-sampling trace remains comparatively continuous, whereas the dark-start mean develops a broad excursion and partial reversal near 36 fs.

**Fig. 7:**
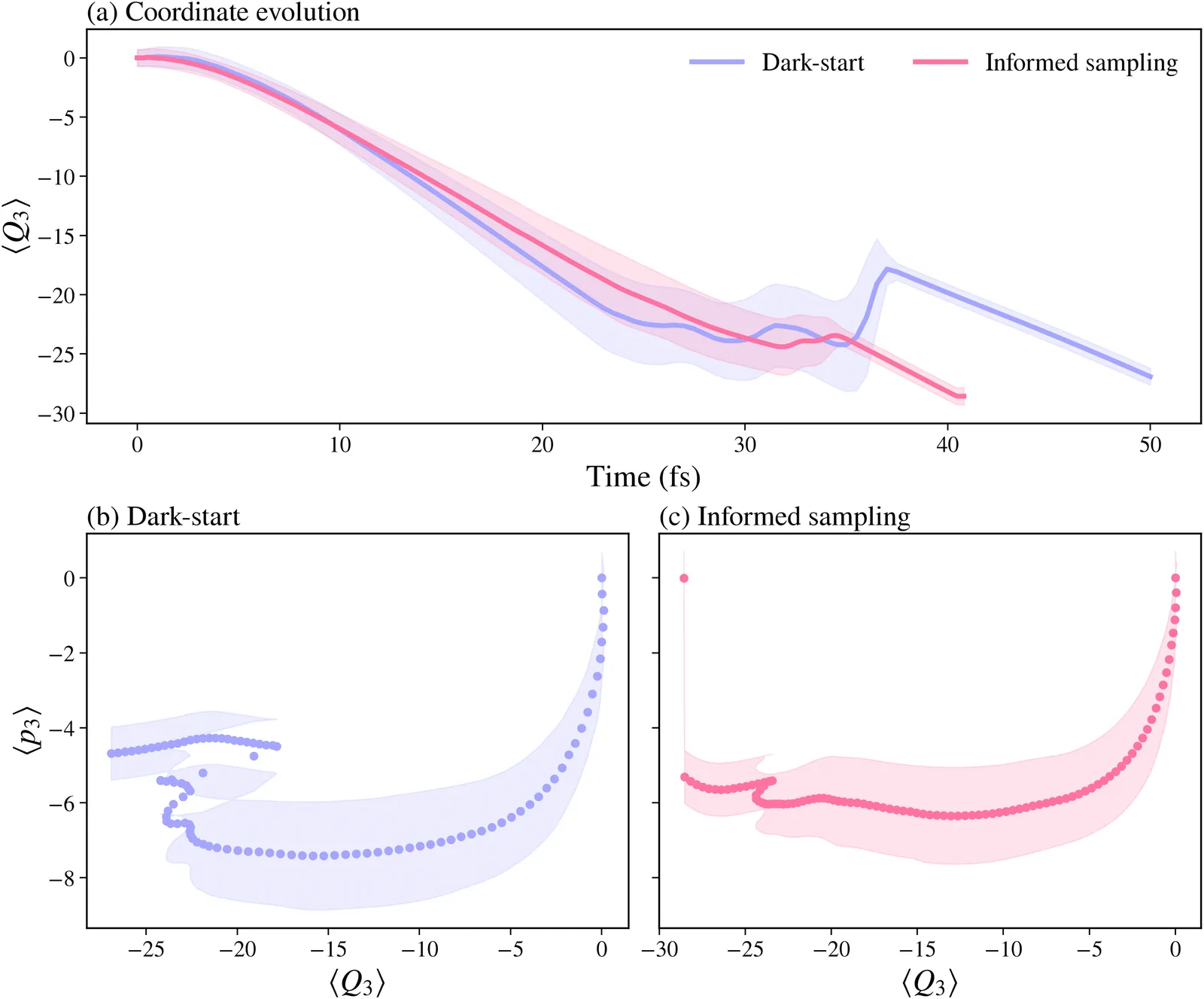
Coordinate and momentum expectation values for the 32-GWP dark-start (blue) and informed-sampling (pink) propagations. (a) Reported coordinate mean 〈*Q*_3_〉 *versus* time, with shading extending coordinate width on either side. (b) and (c) Mean-coordinate–mean-momentum portraits for dark-start and informed sampling, respectively. Points denote successive plotted samples; shading extends one reported momentum width above and below the mean momentum at each sampled mean coordinate. Decreasing *Q*_3_ corresponds to O–O extension, and the CAP begins at *Q*_3_ = −25. Late-time features concern the remaining part of the wavepacket after substantial absorption. Individual unweighted GWP-centre trajectories are shown separately in the SI.

The late-time narrowing of the dark-start band in Fig. 7(a) occurs after most of the wavepacket has been absorbed. At 38 fs, approximately 1.44% of the initial probability remains, and GWP 4 is the only Gaussian with nonzero gross population at the printed precision. The reported coordinate width is then 0.7071, consistent with the surviving amplitude being dominated by a single frozen Gaussian. The narrow band therefore concerns the small remaining part of the wavepacket.

The unweighted centre trajectories provide a compact first-passage statistic for the CAP boundary (SI Fig. S6). The median time at which an individual centre first reaches *Q*_3_ ≤ −25 is 25.75 fs (IQR = 4.13 fs) for the dark-start workflow and 27.30 fs (IQR = 4.95 fs) for informed-sampling. Here, IQR denotes the interquartile range, defined as the difference between the 75th and 25th percentiles of the centre-arrival times. The 1.55 fs difference is smaller than either centre-wise IQR and does not establish a resolved change in dissociation time. Moreover, the statistic assigns equal weight to every centre and neglects interference, so it characterises basis arrival at the CAP rather than population-weighted molecular flux.

The two mean paths follow the same broad phase-space progression (Fig. 7(b and c)). Starting near (*Q*_3_,*p*_3_) = (0,0), both acquire increasingly negative *p*_3_ as the coordinate expectation values move towards O–O extension. The momentum magnitude reaches its largest value at intermediate extension and then decreases as the paths approach the CAP boundary. On the first approach to *Q*_3_ ≈ −25, the informed-sampling mean has a less negative momentum (*p*_3_ ≈ −5.5 to −6) than the dark-start mean (*p*_3_ ≈ −6.5 to −7). The broad, overlapping momentum-width bands make this a descriptive difference rather than a quantitative estimate of kinetic energy.

The late-time structure of the dark-start phase-space portrait concerns the residual amplitude after CAP absorption, which is dominated by GWP 4 at 38 fs, as discussed above. The informed-sampling portrait remains more continuous but retains a smaller kink in the strongly extended region. Before substantial absorption, both portraits show similar O–O extension; the clearer dynamical differences lie in the timing and state distribution of electronic population transfer.

### 4.4 Adiabatic populations and provisional channel analysis

The quasi-diabatic populations in Fig. 6 describe amplitude redistribution in the basis used for propagation. To relate that redistribution to the energy-ordered electronic states and to compare the two workflows using a common channel definition, we additionally project the wavefunction onto the adiabatic manifold. This distinction is important because the quasi-diabatic populations depend on the constructed gauge, whereas the geometry-dependent adiabatic projection introduces a separate numerical approximation.

Fig. 8 (top row) reports adiabatic populations for *S*_0_–*S*_7_ obtained by post-processing the quasi-diabatic wavefunction with geometry-dependent adiabatic projectors. The projector onto adiabatic state *i* is constructed from **C**(***Q***) as23
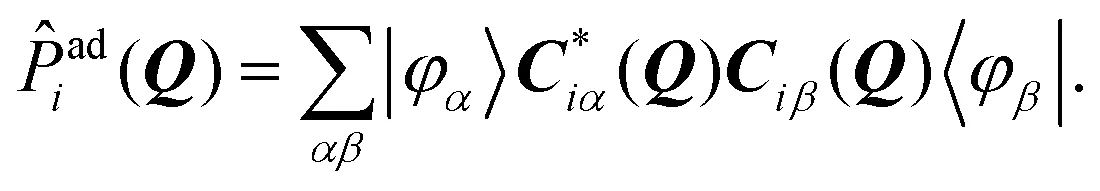


**Fig. 8:**
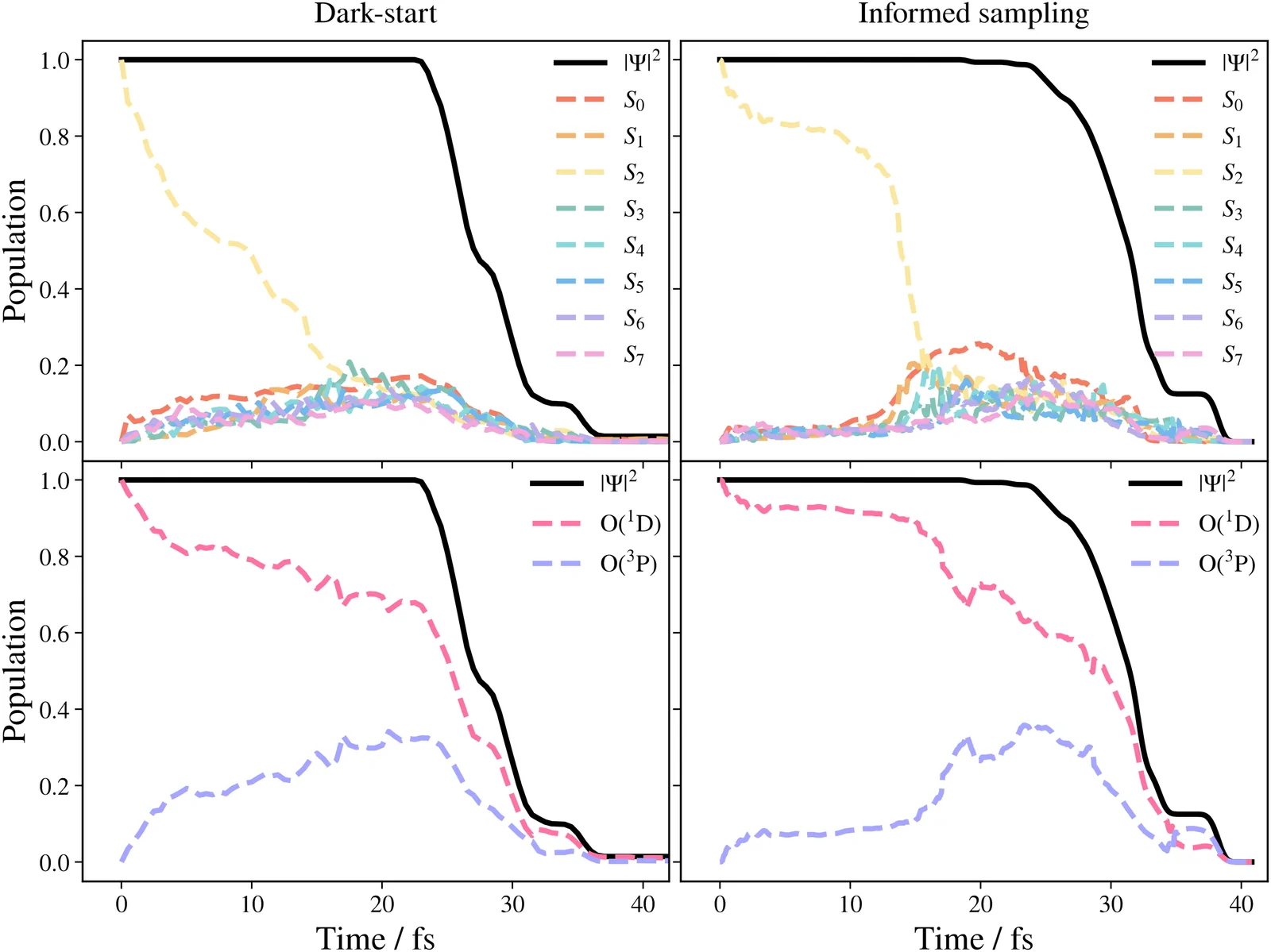
Time evolution of the total wavefunction norm |Ψ(*t*)|^2^ (black solid curves) and absolute projected electronic populations (coloured dashed curves) during DD-vMCG propagation with a CAP. Top row: adiabatic populations *S*_0_–*S*_7_, evaluated from geometry-dependent projectors using the right-hand side saddle point approximation. Bottom row: population sums formed by grouping the adiabatic states according to their asymptotic correlation along the O–O reference scan, labelled O(^1^D) and O(^3^P). Left: Dark-start workflow. Right: Informed-sampling workflow. The initial excitation is to *S*_2_ at the FC geometry. The grouped populations continue to evolve before and during CAP absorption and are therefore qualitative channel-correlation diagnostics, not asymptotic branching yields.

In principle, evaluating 〈Ψ|*P̂*^ad^_*i*_(***Q***)|Ψ〉 requires multidimensional nuclear integration because **C**(***Q***) varies across configuration space. We instead use the right-hand-side saddle-point approximation (invoked by saddle0 in the Quantics program adpop). For a matrix element involving bra GWP *j* and ket GWP *k*, the ADT matrix is evaluated at the centre ***Q***_*k*_ of the ket-side GWP, while the overlap factor *S*_*jk*_ is retained. The approximation therefore retains inter-GWP overlap and interference contributions, but replaces the geometry-dependent projector across the GWP-overlap region by its value at ***Q***_*k*_. The approximation is least reliable when appreciable GWP amplitude spans regions of rapidly varying **C**(***Q***), particularly near conical intersections; further limitations are discussed in SI Section S1.2. Thus, the resulting populations are interpreted as comparative indicators rather than converged observables.

Both workflows transfer population out of the initially prepared *S*_2_ state before substantial CAP loss, but with different temporal profiles (Fig. 8, top row). In the dark-start calculation, *S*_2_ decreases from the beginning and population accumulates gradually across several adiabatic states. With informed-sampling, *S*_2_ remains near 0.8 through approximately 8 fs; its steepest depletion occurs over approximately 13–16 fs, followed by continued redistribution through about 20 fs. The largest receiving component over this interval is *S*_0_, which reaches approximately 0.25, while several excited states each acquire smaller populations. Thus, the principal redistribution is more temporally localised in the informed-sampling calculation, although it remains distinctly multistate.

Previous full-dimensional surface-hopping calculations following vertical excitation to *S*_2_ provide a qualitative comparison of population-transfer times. Antwi *et al.* report depletion beginning at approximately 8 fs, transfer to *S*_1_ over 8–15 fs and transfer involving *S*_3_/*S*_4_ over 10–20 fs.^28^ In Fig. 4 of Esposito *et al.*, the *S*_2_ population falls substantially over approximately 10–20 fs, with *S*_1_ and *S*_0_ as the principal early receiving states.^27^ Both studies retained seven singlet states, compared with eight here. Sit *et al.* constructed potentials and couplings for eight singlet states but restricted FSSH dynamics to O–O extension at separately fixed C–O–O angles. In their decoherence-corrected calculation at 117.7°, Fig. 4(b) shows substantial *S*_2_ depletion over approximately 5–12 fs, with *S*_4_ an important early receiving state.^52^ This occurs earlier than the principal 13–16 fs depletion interval found here. Differences in electronic-structure treatment, initial preparation, nuclear dimensionality and implementation prevent these comparisons from isolating the effect of quantum nuclear propagation.

Over approximately 13–20 fs, the coordinate expectation value in Fig. 7(a) moves from about *Q*_3_ = −7 to −16, passing through and beyond the CoIn2 region identified from the Hamiltonian cuts. The rapid adiabatic population redistribution therefore coincides with the coordinate expectation value traversing this interaction region.

For a provisional channel analysis, we group the adiabatic populations according to their asymptotic correlation along the O–O dissociation scan,24
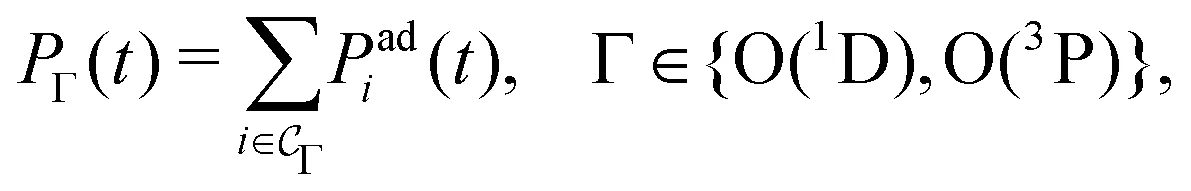
where 
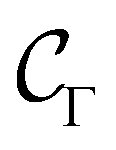
 is the set of adiabatic states correlated with channel Γ on the one-dimensional reference scan. This grouping places the two workflows on the same analytical footing. The informed-sampling quasi-diabatic states permit a more rigorous asymptotic analysis (evaluation of the flux through the CAP operator), but the dark-start labels are insufficiently stable to apply the same direct quasi-diabatic assignment unambiguously. Grouping the projected adiabatic populations therefore supplies a common, albeit approximate, channel descriptor.

If the channel populations reached a plateau before absorption, an instantaneous population-sum estimate,25
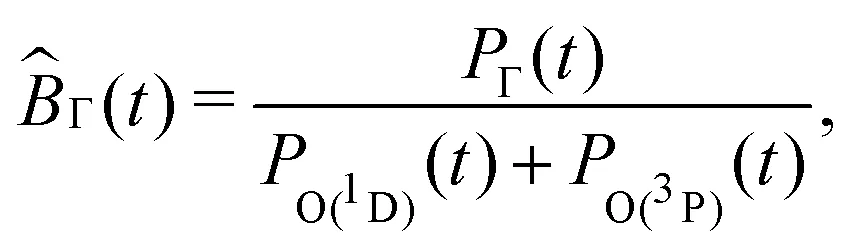
could provide a branching ratio estimate. That condition is not met here. The O(^1^D)- and O(^3^P)-correlated populations continue to change as the CAP begins to remove amplitude, so the bottom row of Fig. 8 supports qualitative trends only. In both workflows the O(^1^D)-correlated sum remains larger, while a substantial O(^3^P)-correlated component develops before the principal norm loss. In the dark-start calculation, the O(^3^P) component grows from the beginning and reaches approximately 0.3–0.35 through an irregular sequence of changes around 18–23 fs. With informed-sampling, it remains near 0.1 for longer, rises sharply during the *S*_2_ depletion interval, and reaches a similar maximum of approximately 0.35 around 20–25 fs. The channel redistribution in the informed-sampling is concentrated more clearly around the interval that includes CoIn2, whereas the dark-start projection evolves earlier and less regularly. Some of this irregularity may reflect the less stable dark-start state correlation and the approximate projector evaluation, so it should not be assigned entirely to physical dynamics.

The grouped populations therefore provide qualitative channel-correlation diagnostics. Quantitative yields require channel-resolved CAP-flux analysis, in which the outgoing amplitude absorbed in each asymptotic manifold is integrated, together with convergence of the electronic manifold, GWP basis, database and projector treatment. For the present workflow comparison, the population sums remain useful because they avoid imposing an ambiguous dark-start quasi-diabatic channel assignment and show how improved state correlation changes the interpretability, rather than the demonstrated value, of a provisional branching analysis.

### 4.5 Additional numerical and spectral comparisons

We additionally examined energy drift and adaptive integration effort in propagation runs with read–write databases. Over the first 20 fs, before appreciable CAP absorption, the maximum absolute energy drift, Δ*E*(*t*) = *E*(*t*) − *E*(0), was 1.06 eV for informed sampling and 2.01 eV for dark-start (corresponding to arithmetic max|Δ*E*|/*N*_G_ estimates of 0.033 and 0.063 eV per GWP, respectively, for *N*_G_ = 32). During the post-startup interval of 0.5–20 fs, the informed calculation required 1172 attempted RK5 steps, compared with 2148 for dark-start. The complete diagnostic definitions, integration statistics and eight-state electronic-energy prediction errors at the geometries requested during propagation are reported in SI Section S9.

Separately, energy-weighted spectral profiles obtained from the wavepacket autocorrelations were compared with the experimental absorption spectra of Ting *et al.* and Mir *et al.*^25,55^ After aligning the wavelength maxima, the informed profile agrees more closely with their broad envelopes over 310–430 nm, although both calculated profiles retain a red tail. The spectra, experimental sources and common processing procedure are provided in SI Section S10 and Fig. S12.

## Conclusions

This study identifies database initialisation as a controllable source of sampling-history dependence in propagation-diabatised DD-vMCG calculations. In a dark-start workflow, the geometries encountered during exploratory propagation become the first references for interpolation and diabatisation, so their order and spatial distribution can condition the resulting quasi-diabatic representation. We addressed this practical dependence by pre-seeding 97 geometries along the dissociation-dominated *Q*_3_ coordinate, spanning the FC region, CoIn1, CoIn2 and stretched O–O configurations, and rediabatising them before database-growth and production dynamics. The anchors are treated with the same updated two-branch predictor-corrector procedure used during production. Informed-sampling therefore changes the initial reference coverage, but neither the vMCG equations nor the underlying propagation-diabatisation formalism.

The one-dimensional CASPT2 and XMS-CASPT2 comparisons show that perturbative correlation changes the absolute dissociation energetics, while preserving the broad division into two asymptotic state groups and the clustering of near-degeneracies along the sampled O–O coordinate. These scans support using SA-CASSCF(10e,8o)/6-31G(d,p) to compare database-initialisation workflows at tractable cost.

Within this benchmark, both workflows show broadly similar O–O extension and population redistribution among states correlated with the two dissociation channels. The informed-sampling, however, produces a smoother and less initialisation-sensitive quasi-diabatic Hamiltonian along *Q*_3_. The initial informed-sampling crossing sequences through CoIn1 and CoIn2 remain recognisable after the database grows to approximately 5 × 10^3^ points, whereas the matched dark-start database retains additional curvature and a less clearly ordered upper-state structure, most prominently around CoIn2. On the common diagnostic grid, the aggregate running RMS curvature decreases from *S*_tot_ (*s*_max_) ≈ 1.64 for the dark-start production database to 0.851 for informed-sampling, a reduction of approximately 48%. The informed-sampling value also remains close to that of its initial database, while the dark-start value increases relative to its *N*_G_ = 32 initial database. Together with the basis-size comparisons in the SI, these results show that subsequent database growth does not necessarily remove structure introduced during early sampling and that targeted pre-seeding reduces this sensitivity for the tested initialisations.

Within the retained eight-state model, informed sampling concentrates the changes in dominant adiabatic character within the resolved interaction windows, with comparatively stable assignments between them. Dark-start gives broader and less consistently correlated changes, particularly in the upper manifold.

Both workflows move towards O–O extension, and their median unweighted GWP-centre first-passage times to the CAP boundary differ by 1.55 fs, less than either centre-wise IQR. Informed sampling retains population in *S*_2_ for longer and concentrates the principal adiabatic redistribution within approximately 13–20 fs, while the coordinate expectation value moves from approximately *Q*_3_ = −7 to −16, through and beyond CoIn2.

The grouped O(^1^D)- and O(^3^P)-correlated populations provide channel diagnostics, with both workflows reaching a maximum O(^3^P)-correlated population of approximately 0.35. These populations do not reach a stationary plateau before CAP absorption, so they do not determine asymptotic branching yields. Quantitative yields will require channel-resolved CAP-flux analysis together with convergence with respect to the GWP basis, database density, anchor-set construction and projector treatment.

To investigate the influence of quantum nuclear motion on CH_2_OO photochemistry, we plan to construct a common XMS-CASPT2 potential-energy database and compare TSH and DD-vMCG propagation within QUANTICS in read-only mode. Following the controlled-comparison strategy discussed in the cyclobutanone perspective,^34^ the comparison will use the same electronic manifold, comparable initial nuclear preparations and consistent population definitions, with checks of numerical convergence and database coverage.

## Author contributions

KM was responsible for the investigation, formal analysis, data curation, visualisation and writing of the original draft. GW was responsible for the conceptualisation, project management and providing the resources required. Both KM and GW were responsible for the development of the methodology and software used, as well as the validation of results and reviewing and editing the final draft.

## Conflicts of interest

There are no conflicts to declare.

## Supplementary Material

CP-028-D6CP02996E-s001

## Data Availability

The data from the calculations is available from the UCL Research Data Repository at DOI: https://doi.org/10.5522/04/33111968. This includes the input and output files, along with the final quantum chemistry databases, from the two workflows. The python code used in the analysis to map the O–O distance along the reference cut onto the database geometries is also available from that site. The Quantics suite is available on request. See https://www.chem.ucl.ac.uk/quantics for details. Supplementary information: details of analysis and observables calculated; mapping the O–O reference path onto database geometries; sensitivity of the initial quasi-diabatic Hamiltonian; complete adiabatic-composition weights; unweighted GWP-centre trajectories; state-resolved initial database curvature; additional normal-coordinate cuts; energy prediction and numerical propagation with read–write databases; spectral-envelope comparison. See DOI: https://doi.org/10.1039/d6cp02996e.
