# Supplementary material for: Pre-seeding diabatic anchors for reproducible direct-dynamics quantum photodissociation: an 8-state full-dimensional DD-vMCG study of CH2OO
Source: Phys Chem Chem Phys. 2026 Sep 29;28(41):25748–63. doi: 10.1039/d6cp02996e (PMC13643721; doi:10.1039/d6cp02996e)
Supplement: Supplementary file 1 [file CP-028-D6CP02996E-s001.pdf]

Supplementary Information (SI) for Physical Chemistry Chemical Physics.
This journal is © the Owner Societies

Supporting Information: Pre-seeding diabatic anchors for reproducible direct-dynamics quantum photodissociation: An 8-state full-dimensional DD-vMCG study of CH_2OO

Kevin Ma and Graham A. Worth*

September 25, 2026

Dept. of Chemistry, University College London, 20, Gordon St., London, WC1H 0AJ,
* g.a.worth@ucl.ac.uk.

Contents

1 Observables and analysis 3
1.1 Electronic-state populations, GGPs and coordinate moments 3
1.2 Adiabatic populations and the RHS Saddle Point approximation 4
1.3 Curvature-based smoothness diagnostic 4
1.4 Electronic-character analysis 5

2 Mapping the O–O reference path onto database geometries 6
2.1 Reference and database coordinates 6
2.2 Configurable distance metric 6
2.3 Global one-to-one assignment 7
2.4 Retrieval and plotting 7

3 Sensitivity of the initial quasi-diabatic Hamiltonian 8

4 Complete adiabatic-composition weights 10

5 Unweighted GWP-centre trajectories 12

6 Dark-start $\langle Q_3 \rangle$ vs time with different number of GWPs 14

7 State-resolved initial-database curvature 15

8 Additional normal-coordinate cuts **16**

9 Energy prediction and numerical propagation with read–write databases **18**

10 Spectral-envelope comparison **20**

11 Computational protocol and cost accounting **21**

11.1 Database construction, Hessian updating and interpolation 21

11.2 Local cubic correction 21

11.3 Electronic-structure cost 22

1 Observables and analysis

1.1 Electronic-state populations, GGPs and coordinate moments

For the single-set DD-vMCG expansion,

$$|\Psi(t)\rangle = \sum_{\alpha=1}^{N_{\rm st}} \sum_{k=1}^{N_G} A_k^{(\alpha)}(t)\, |g_k(t)\rangle\, |\varphi_\alpha\rangle\,, \tag{1}$$

the absolute population of quasi-diabatic state α is

$$\begin{aligned} P_\alpha^{\rm qd}(t) =& \ \langle \Psi(t)|\hat{P}_\alpha^{\rm qd}|\Psi(t)\rangle \\ =& \sum_{k,l=1}^{N_G} A_k^{(\alpha)*}(t) S_{kl}(t) A_l^{(\alpha)}(t), \qquad \hat{P}_\alpha^{\rm qd} = |\varphi_\alpha\rangle\,\langle\varphi_\alpha|\,, \end{aligned} \tag{2}$$

where $S_{kl}(t) = \langle g_k(t)|g_l(t)\rangle$ is the GWP overlap matrix. The sum over α gives the instantaneous wavefunction norm. Equation (2) contains both diagonal and off-diagonal GWP-overlap contributions and is therefore not a count of independent GWP trajectories.

To associate a contribution with each non-orthogonal GWP, we use the Mulliken-like gross Gaussian population (GGP) analysis,[1]

$$G_k^{(\alpha)}(t) = \mathrm{Re}\left[A_k^{(\alpha)*}(t) \sum_{l=1}^{N_G} S_{kl}(t) A_l^{(\alpha)}(t) \right], \tag{3}$$

$$G_k(t) = \sum_{\alpha=1}^{N_{\rm st}} G_k^{(\alpha)}(t). \tag{4}$$

The decomposition satisfies

$$\sum_{k=1}^{N_G} G_k(t) = \langle \Psi(t)|\Psi(t)\rangle. \tag{5}$$

An individual G_k is a signed partition of the norm rather than a probability: off-diagonal overlap terms can contribute constructively or destructively and can, in principle, give a negative gross population.

For $\mathcal{N}(t) = \langle\Psi(t)|\Psi(t)\rangle > 0$, the normalised coordinate moments are

$$\langle Q_3^r\rangle(t) = \frac{\sum_{\alpha,j,k} A_j^{(\alpha)*}(t) A_k^{(\alpha)}(t) \langle g_j(t)|\hat{Q}_3^r|g_k(t)\rangle}{\mathcal{N}(t)}, \qquad r = 1, 2, \tag{6}$$

with coordinate width

$$\Delta Q_3(t) = \sqrt{\langle Q_3^2\rangle(t) - \langle Q_3\rangle(t)^2}. \tag{7}$$

These matrix elements include the finite Gaussian widths and inter-GWP contributions. The plotting script reads the mean and width for mode `v3` from the Quantics output fields `<q>` and `<dq>` and plots one reported width on either side of the mean in main-text Fig. 7(a). Momentum moments are defined analogously with $\hat{p}_3$ in place of $\hat{Q}_3$. The phase-space portraits in Fig. 7(b,c) plot the reported coordinate expectation value against the reported momentum expectation value, with vertical shading extending one reported momentum width on either side. They are not joint coordinate–momentum probability distributions.

1.2 Adiabatic populations and the RHS Saddle Point approximation

The geometry-dependent projector onto adiabatic state i is

$$\begin{aligned}\hat{P}_i^{\mathrm{ad}}(\boldsymbol{Q}) &= |\psi_i(\boldsymbol{Q})\rangle \langle\psi_i(\boldsymbol{Q})| \\ &= \sum_{\alpha,\beta} |\varphi_\alpha\rangle\, C_{i\alpha}^*(\boldsymbol{Q}) C_{i\beta}(\boldsymbol{Q}) \langle\varphi_\beta| \,.\end{aligned} \tag{8}$$

Exact evaluation of $P_i^{\mathrm{ad}}(t) = \langle\Psi(t)|\hat{P}_i^{\mathrm{ad}}(\boldsymbol{Q})|\Psi(t)\rangle$ requires multidimensional nuclear matrix elements because $\mathbf{C}(\boldsymbol{Q})$ varies across configuration space. The populations reported in the main text were instead obtained with the `-saddle0` option of the Quantics `adpop` analysis program.[2] For each GWP k, $\mathbf{C}$ is evaluated at its centre $\boldsymbol{Q}_k$, and applied to overlap matrix elements with the GWP index on the right hand side:

$$P_i^{\mathrm{ad,\,s0}}(t) = \sum_{j,k=1}^{N_G} S_{jk}(t) \sum_{\alpha,\beta} A_j^{(\alpha)*}(t) C_{i\alpha}^*(\boldsymbol{Q}_k) C_{i\beta}(\boldsymbol{Q}_k) A_k^{(\beta)}(t) \tag{9}$$

In comparison to the full saddle-point approximation, the RHS saddle point thus further approximates the operator for off-diagonal matrix elements ($k \neq j$). The saddle point approximation makes an obvious error when A GWP crosses a conical intersection as the integral cannot take into account the change in state character in the region under its envelope. The RHS saddle point is further expected to be least reliable when overlapping GWPs carry appreciable amplitude in regions where $\mathbf{C}(\boldsymbol{Q})$ changes rapidly. The resulting adiabatic populations are consequently used as comparative state-correlation diagnostics rather than converged observables.

1.3 Curvature-based smoothness diagnostic

The displacement from the FC geometry along the dissociative direction is defined as

$$s = Q_3^{\mathrm{FC}} - Q_3, \qquad s \geq 0, \qquad Q_3^{\mathrm{FC}} \approx 0, \tag{10}$$

and each diagonal quasi-diabatic cut is written as

$$E_\alpha(s) = W_{\alpha\alpha}(Q_3^{\mathrm{FC}} - s). \tag{11}$$

The accumulated squared curvature and running root-mean-square (RMS) curvature are

$$I_\alpha(s) = \int_0^s \left[\frac{d^2 E_\alpha(s')}{ds'^2}\right]^2 ds', \tag{12}$$

$$S_\alpha(s) = \sqrt{\frac{I_\alpha(s)}{s}}, \qquad s > 0, \tag{13}$$

with the aggregate diagnostic

$$S_{\mathrm{tot}}(s) = \sum_{\alpha=1}^{N_{\mathrm{st}}} S_\alpha(s). \tag{14}$$

The sampled coordinates are sorted, and energies at duplicate coordinates are averaged. The second derivative is evaluated by applying `numpy.gradient` twice with these coordinates

and `edge_order=2` in each call. The squared values are accumulated by cumulative trapezoidal integration. The running RMS is evaluated for positive displacement and the initial plotted value is set to zero. Both workflows use the same grid and numerical procedure. Only values obtained on the same coordinate interval, grid and interpolation procedure are compared. The metric is coordinate dependent, sensitive to numerical differentiation and responsive to both physical and non-physical curvature. It is therefore a comparative smoothness diagnostic, not a molecular observable or a stand-alone measure of global Hamiltonian accuracy.

1.4 Electronic-character analysis

At a mapped geometry $\boldsymbol{Q}$, the contribution of adiabatic state i to fixed quasi-diabatic state α is

$$w_{i\to\alpha}(\boldsymbol{Q}) = |C_{i\alpha}(\boldsymbol{Q})|^2. \tag{15}$$

The dominant adiabatic index displayed in the right-hand column of the main-text character figure is

$$i_\alpha(\boldsymbol{Q}) = \arg\max_i |C_{i\alpha}(\boldsymbol{Q})|^2. \tag{16}$$

The maximum is evaluated independently for each quasi-diabatic state α. This operation does not impose a one-to-one correspondence between adiabatic and quasi-diabatic indices. Near a strongly mixed or degenerate region, more than one quasi-diabatic state may therefore have the same dominant adiabatic index. The complete weights in Figs. 3 and 4 should be consulted wherever the dominant-index map compresses such mixing into a single colour.

2 Mapping the O–O reference path onto database geometries

The character analysis requires ADT matrices and diagonal quasi-diabatic potentials at database geometries corresponding as closely as possible to a common O–O dissociation path. Reference path geometries were therefore matched to distinct geometries in each production SQLite database. Here, a global minimum-cost one-to-one assignment is implemented.

2.1 Reference and database coordinates

The reference path contains N_{ref} geometries with increasing O–O bond length, represented in the same $f = 9$ rectilinear mass- and frequency-weighted normal coordinates used for the dynamics,

$$\boldsymbol{Q}_i^{\mathrm{ref}} = \left(Q_{i,1}^{\mathrm{ref}}, \ldots, Q_{i,f}^{\mathrm{ref}}\right) \in \mathbb{R}^f. \tag{17}$$

The points are ordered along Q_3, the normal mode coordinate dominated by O–O stretching. Their corresponding Cartesian structures provide the O–O distance $R_{\mathrm{OO},i}$ used as the horizontal axis of the character plots. Unlike the pure-Q_3 seed line, this reference path displaces Q_1, Q_3, Q_5, Q_6, Q_7, Q_8 and Q_9. It is therefore a one-dimensional, multi-coordinate curve away from the seed line. The character analysis matches this curve to geometries already present in each production database; it is not a held-out quantum-chemistry test.

The target production database contains M geometries,

$$\boldsymbol{Q}_j^{\mathrm{db}} = \left(Q_{j,1}^{\mathrm{db}}, \ldots, Q_{j,f}^{\mathrm{db}}\right) \in \mathbb{R}^f, \qquad j = 1, \ldots, M, \tag{18}$$

stored in the `nmode` table together with unique integer IDs. The stored IDs link each coordinate vector to the corresponding entries in the `trans` and `pes` tables.

2.2 Configurable distance metric

For reference point i and database point j, we construct the squared cost

$$c_{ij} = \sum_{\mu=1}^{f} w_\mu \left[s_\mu \left(Q_{i,\mu}^{\mathrm{ref}} - Q_{j,\mu}^{\mathrm{db}} \right) \right]^2. \tag{19}$$

Current implementation supports either unscaled coordinates, $s_\mu = 1$, or sample-standard-deviation scaling,

$$s_\mu = \sigma_\mu^{-1}, \qquad \sigma_\mu = \left[\frac{1}{M-1} \sum_{j=1}^{M} \left(Q_{j,\mu}^{\mathrm{db}} - \overline{Q}_\mu^{\mathrm{db}} \right)^2 \right]^{1/2}. \tag{20}$$

If a standard deviation is smaller than the numerical threshold, its scale factor is reset to unity. The optional weights are $w_\mu = 1$ by default. A selected mode can be prioritised by multiplying its quadratic penalty by a factor $\lambda \geq 1$; for Q_3, this corresponds to $w_3 \leftarrow \lambda w_3$. The database wrapper supplied with the analysis defaults to unscaled, equally weighted coordinates unless non-default `scale_mode`, `priority_mode` and `priority_scale` arguments are passed.

2.3 Global one-to-one assignment

Independent nearest-neighbour selection can map several reference geometries to the same database structure. Distinct matches are instead obtained by solving

$$\min_{\pi} \sum_{i=1}^{N_{\mathrm{ref}}} c_{i,\pi(i)} \qquad \text{subject to } \pi \text{ being injective,} \tag{21}$$

This linear-sum assignment problem is solved using SciPy's `linear_sum_assignment` function.[3, 4] The condition $M \geq N_{\mathrm{ref}}$ permits every reference point to receive a distinct database index. Equation (21) minimises the total cost over all matches. The assigned point for a particular reference geometry is therefore not guaranteed to be that geometry's independent minimum-cost target under Eq. (19).

The current implementation records the independent minimum-cost ID, the assigned ID, the scaled and unscaled distances, and the rank of the assigned point within the cost-ordered candidates for each reference geometry. A rank of zero indicates that the uniqueness constraint did not displace the independent minimum-cost match. These diagnostics allow unusually non-local assignments to be identified rather than hidden by the global cost alone.

2.4 Retrieval and plotting

After assignment, the selected IDs are used to retrieve the ADT matrices from the `trans` table and the diagonal quasi-diabatic potentials from the `pes` table. The database columns are read in column-major order and transposed after reshaping so that the resulting arrays have indices $[\mathrm{geometry}, i, \alpha]$. The rows returned by SQLite are explicitly reordered to match the assigned ID sequence.

For the main-text figure, the complete composition array is $|C_{i\alpha}|^2$, and Eq. (16) is evaluated over the adiabatic index i. The resulting path axis is reversed only as required to align the mapped data with increasing R_{OO} in the displayed image; this plotting operation does not reassign either the database geometries or the electronic-state labels. The left-hand panels plot the corresponding diagonal potentials, while the right-hand panels plot the independently selected dominant adiabatic index for each fixed quasi-diabatic state.

3 Sensitivity of the initial quasi-diabatic Hamiltonian

Figure 1 shows *dark-start* initialisations generated with different GWP basis sizes. Figure 2 shows the corresponding *informed-sampling* calculations, all of which begin from the same initial database containing the FC entry and 97 additional dissociation-path seed entries. The collection tests sensitivity to the sampled initialisation; it is not, by itself, a convergence series in N_G.

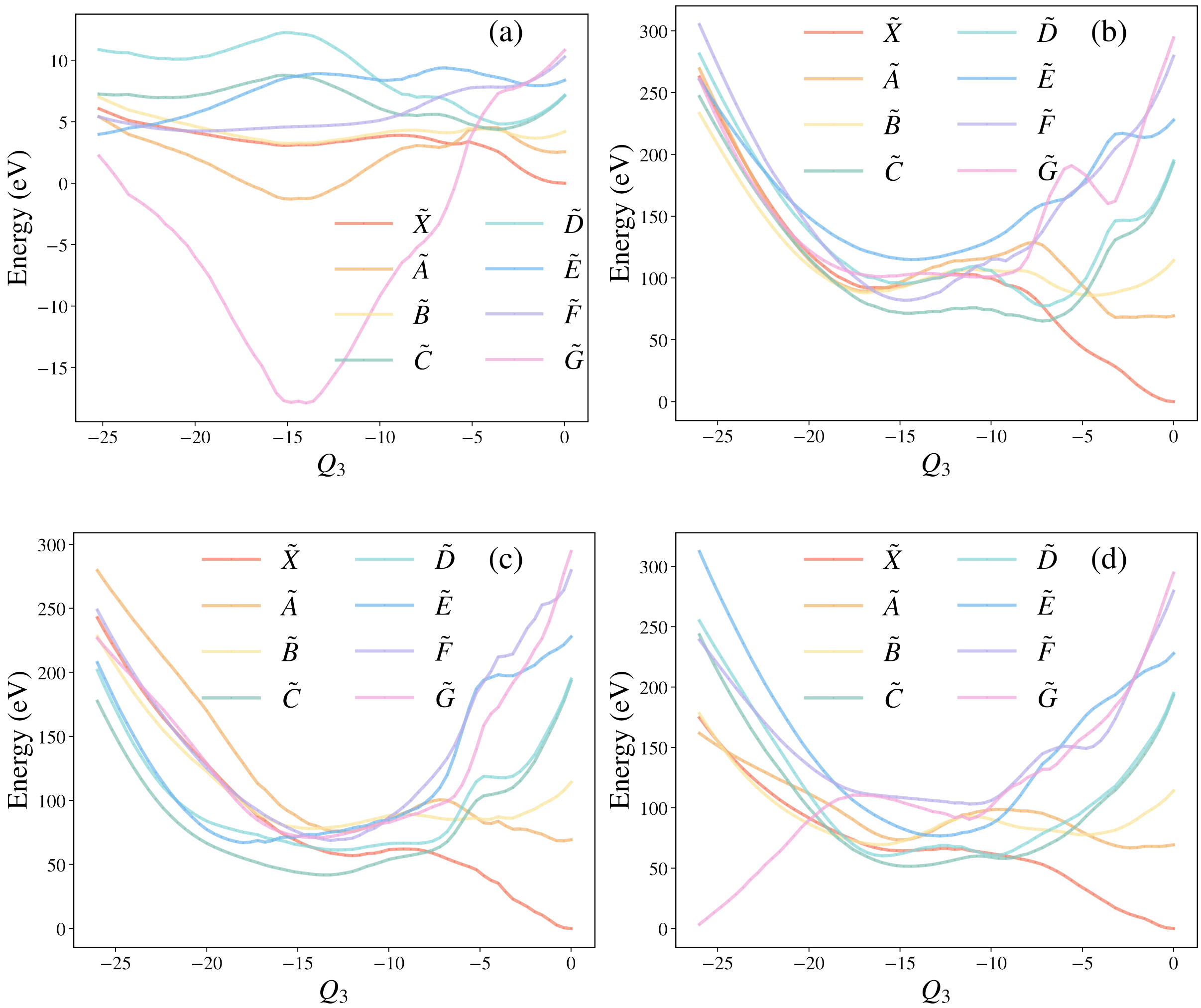

Figure 1: One-dimensional cut of the initial diagonal quasi-diabatic potentials $W_{\alpha\alpha}(Q_3)$ obtained from *dark-start* initialisation with (a) $N_G = 4$, (b) $N_G = 19$, (c) $N_G = 28$ and (d) $N_G = 32$. All other normal coordinates are fixed at their FC values, and decreasing Q_3 corresponds to O–O extension. Energies are reported relative to the ground-state energy at the FC geometry.

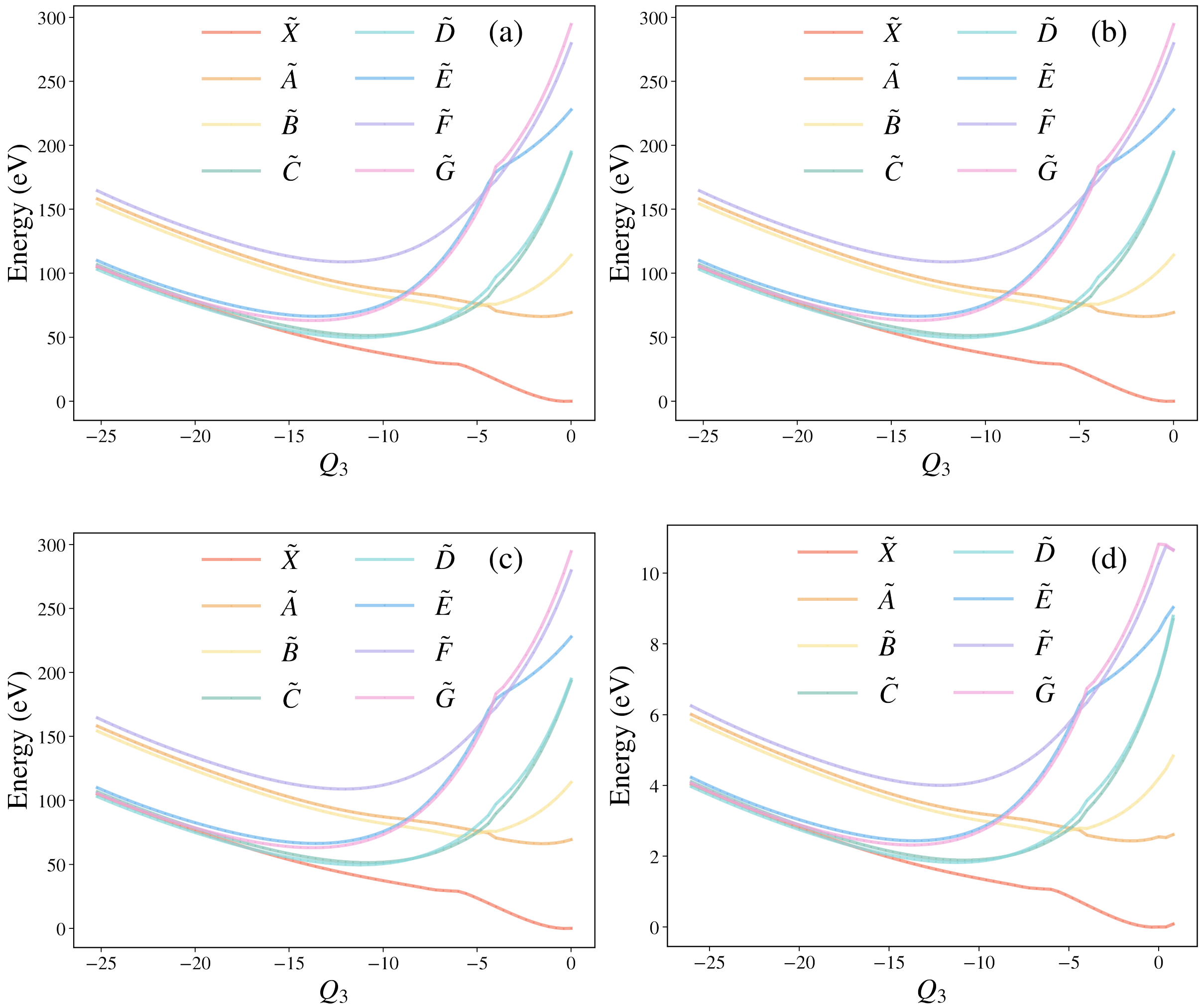

Figure 2: One-dimensional cut of the initial diagonal quasi-diabatic potentials $W_{\alpha\alpha}(Q_3)$ obtained from *informed-sampling* calculations initialised with the same 98-entry database
, with (a) $N_G = 4$, (b) $N_G = 19$, (c) $N_G = 28$ and (d) $N_G = 32$. All other normal coordinates are fixed at their FC values, and decreasing Q_3 corresponds to O–O extension. Energies are reported relative to the ground-state energy at the FC geometry.

4 Complete adiabatic-composition weights

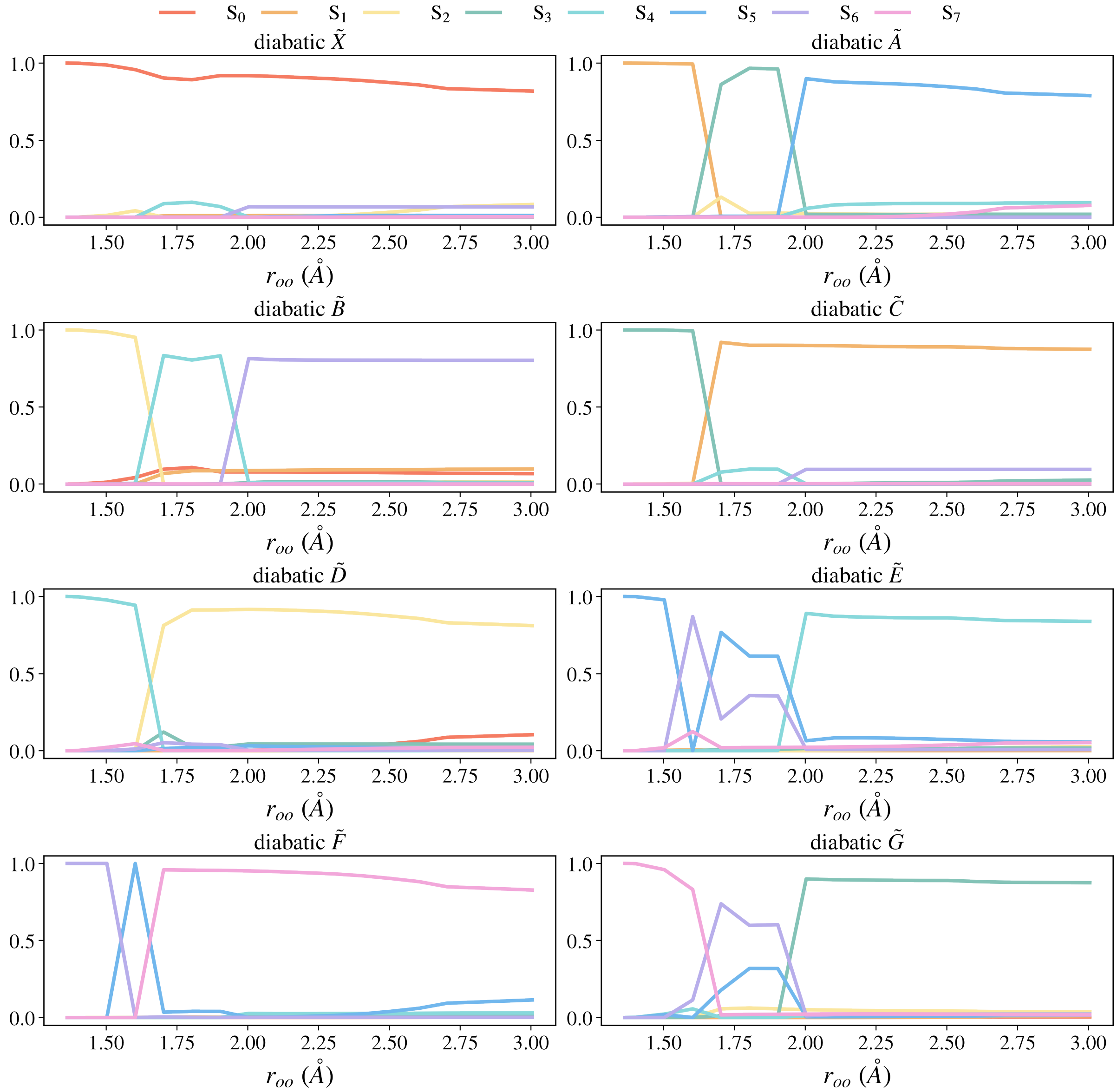

Figure 3: Adiabatic-composition weights $w_{i\to\alpha}(R_{\mathrm{OO}}) = |C_{i\alpha}(R_{\mathrm{OO}})|^2$ for the *informed-sampling* production database. Each panel corresponds to one fixed quasi-diabatic state $\alpha \in \{\tilde{X}, \tilde{A}, \ldots, \tilde{G}\}$; coloured curves denote the contributions from adiabatic states S_0–S_7. The ADT matrices are evaluated at the distinct database geometries obtained from the global one-to-one mapping described in Sec. 2. These complete weights underlie the dominant-index map in the top-right panel of the corresponding main-text figure.

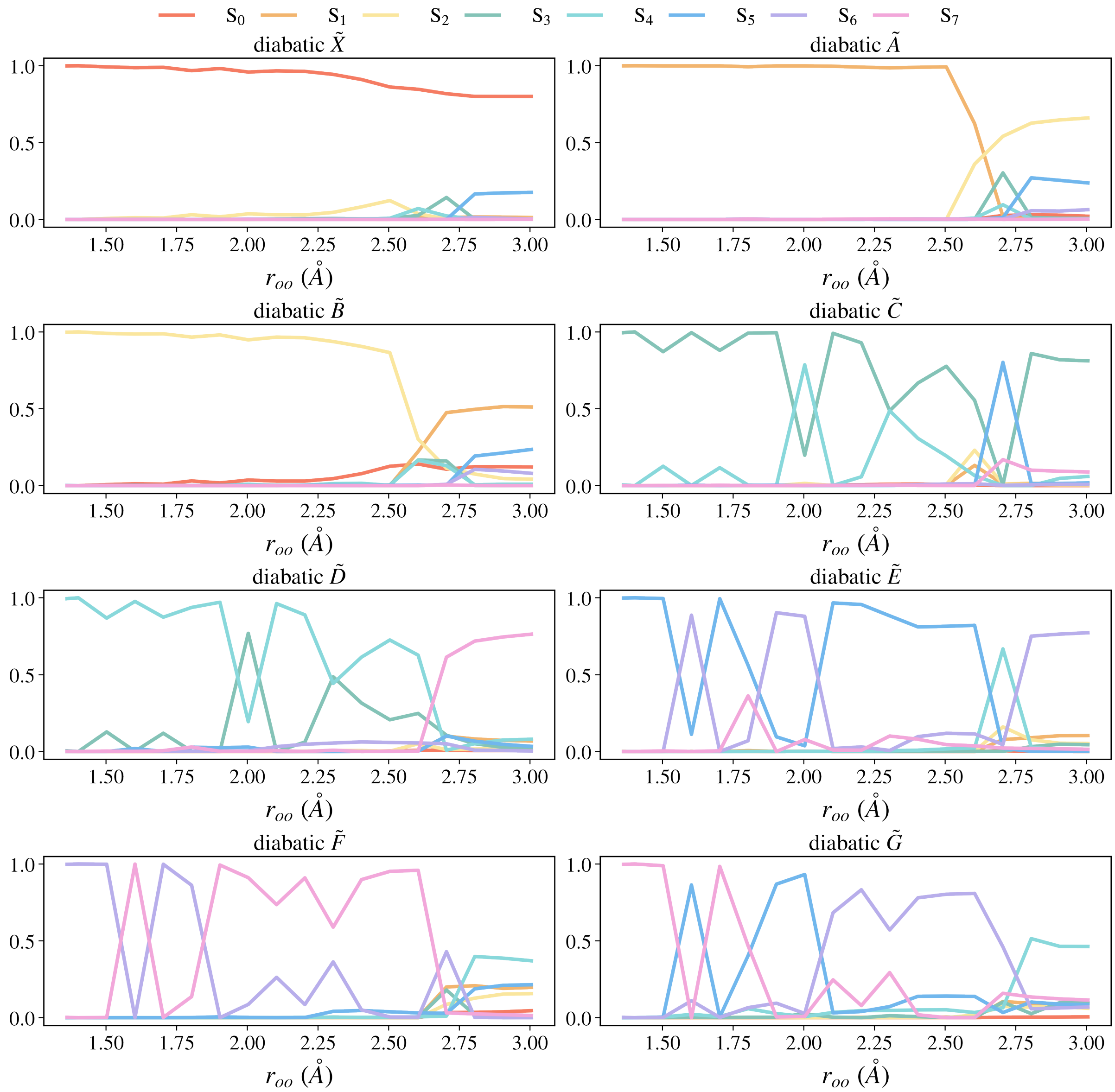

Figure 4: Adiabatic-composition weights $w_{i\to\alpha}(R_{\rm OO}) = |C_{i\alpha}(R_{\rm OO})|^2$ for the *dark-start* production database. Each panel corresponds to one fixed quasi-diabatic state $\alpha \in \{\tilde{X}, \tilde{A}, \ldots, \tilde{G}\}$; coloured curves denote the contributions from adiabatic states S_0–S_7. The ADT matrices are evaluated at the distinct database geometries obtained from the global one-to-one mapping described in Sec. 2. These complete weights underlie the dominant-index map in the bottom-right panel of the corresponding main-text figure.

5 Unweighted GWP-centre trajectories

The centre-arrival statistic in the main text uses the first time each GWP centre reaches $Q_3 \leq -25$, the CAP onset. Its interquartile range (IQR) is the difference between the 75th and 25th percentiles of these arrival times. Each centre has equal weight in this statistic; it measures basis arrival rather than population-weighted molecular flux.

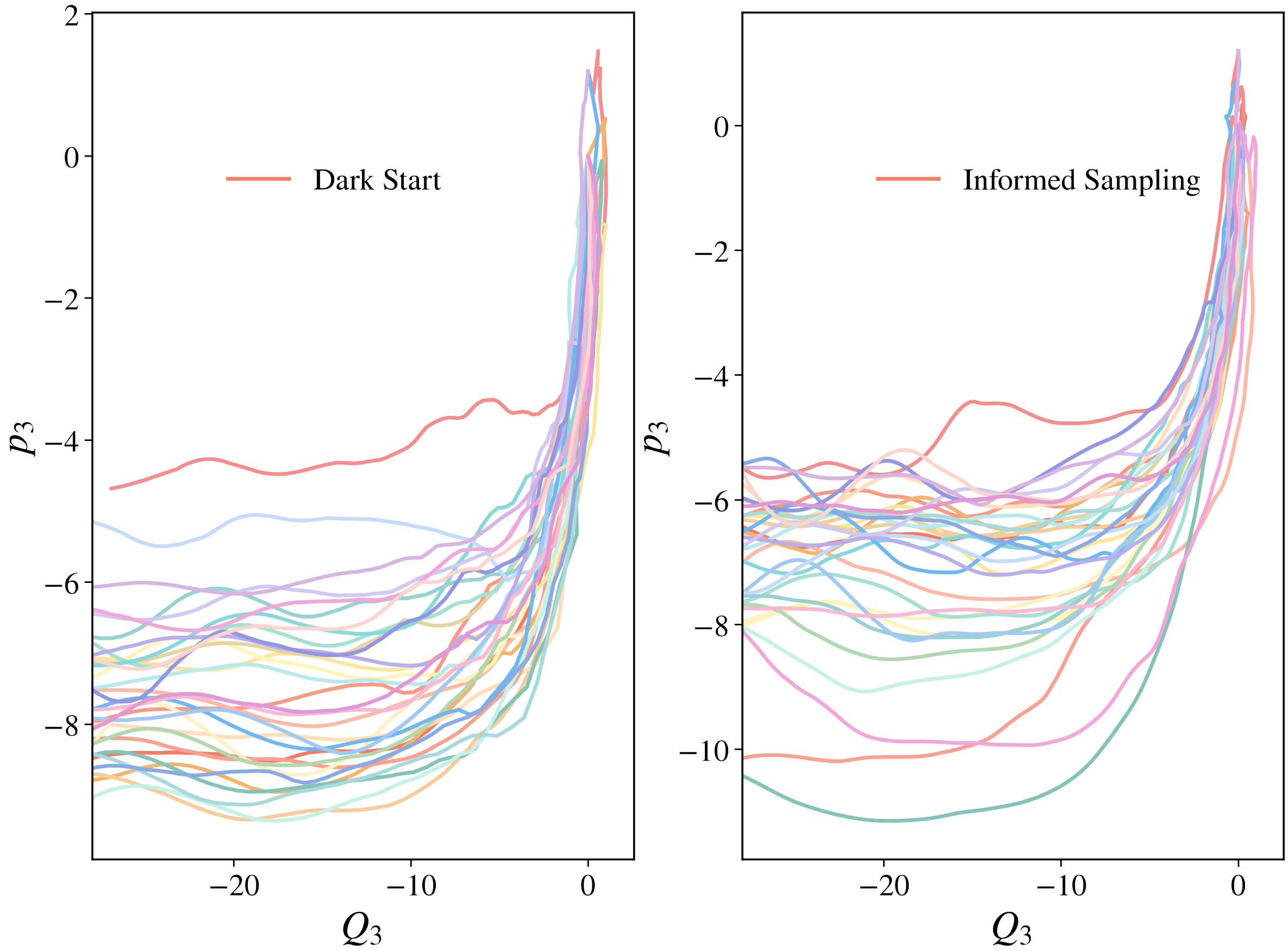

Figure 5: Individual GWP-centre trajectories projected onto the (Q_3, p_3) phase plane for the $N_G = 32$ DD-vMCG propagations. Left: *dark-start* workflow. Right: *informed-sampling* workflow. Each coloured curve follows one GWP centre and carries no population weight. The trajectories therefore expose the dispersion of the finite basis but do not represent independent classical paths or channel-resolved flux.

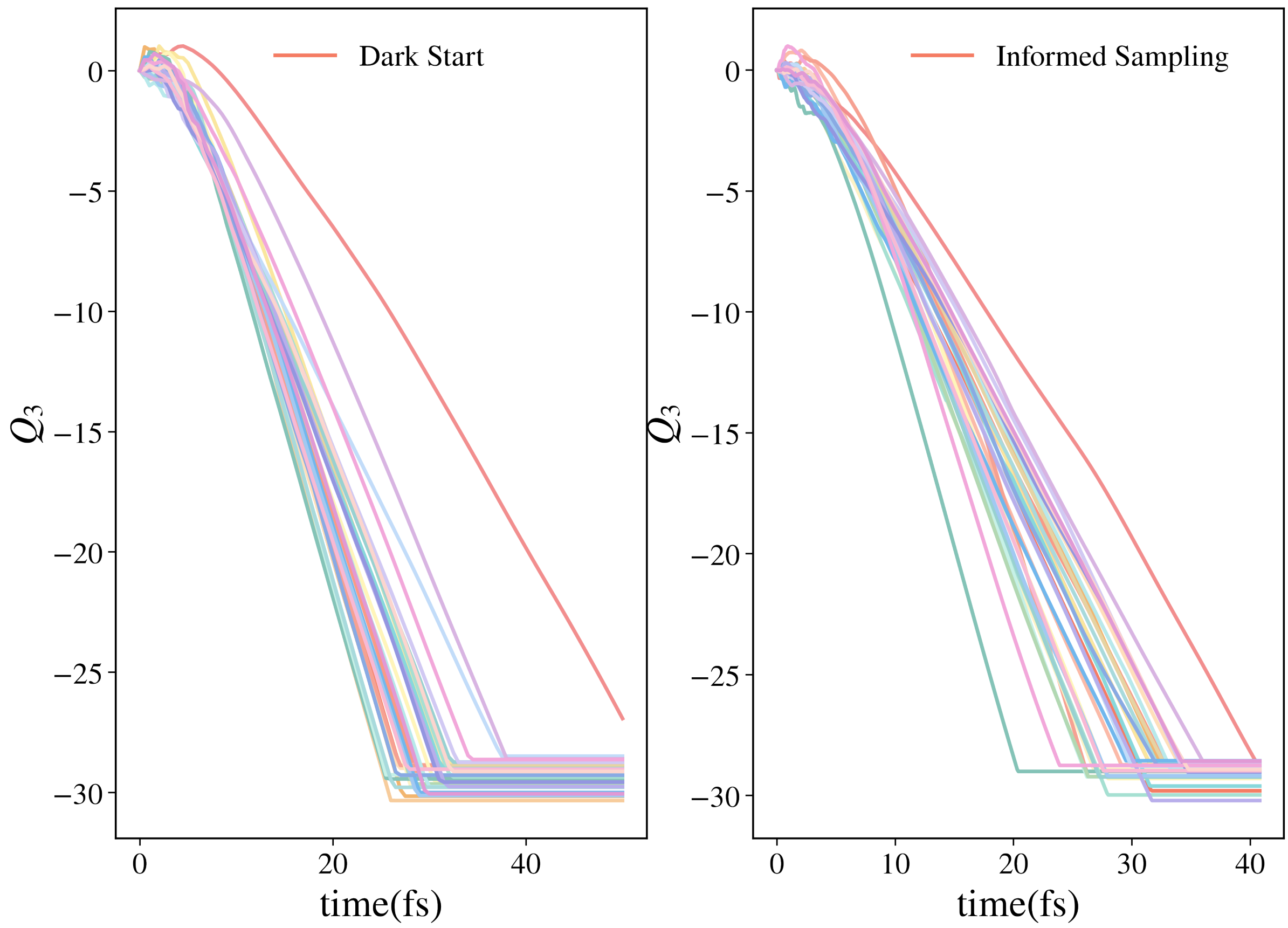

Figure 6: Time evolution of the individual GWP centres along the O–O-stretch-dominated coordinate Q_3 for the $N_G = 32$ DD-vMCG propagations. Left: *dark-start* workflow. Right: *informed-sampling* workflow. Each coloured curve follows one centre without GGP weighting. Decreasing Q_3 corresponds to O–O extension; the nearly horizontal late-time segments occur in the CAP region and should not be interpreted as stationary product populations.

6 Dark-start $\langle Q_3 \rangle$ vs time with different number of GWPs

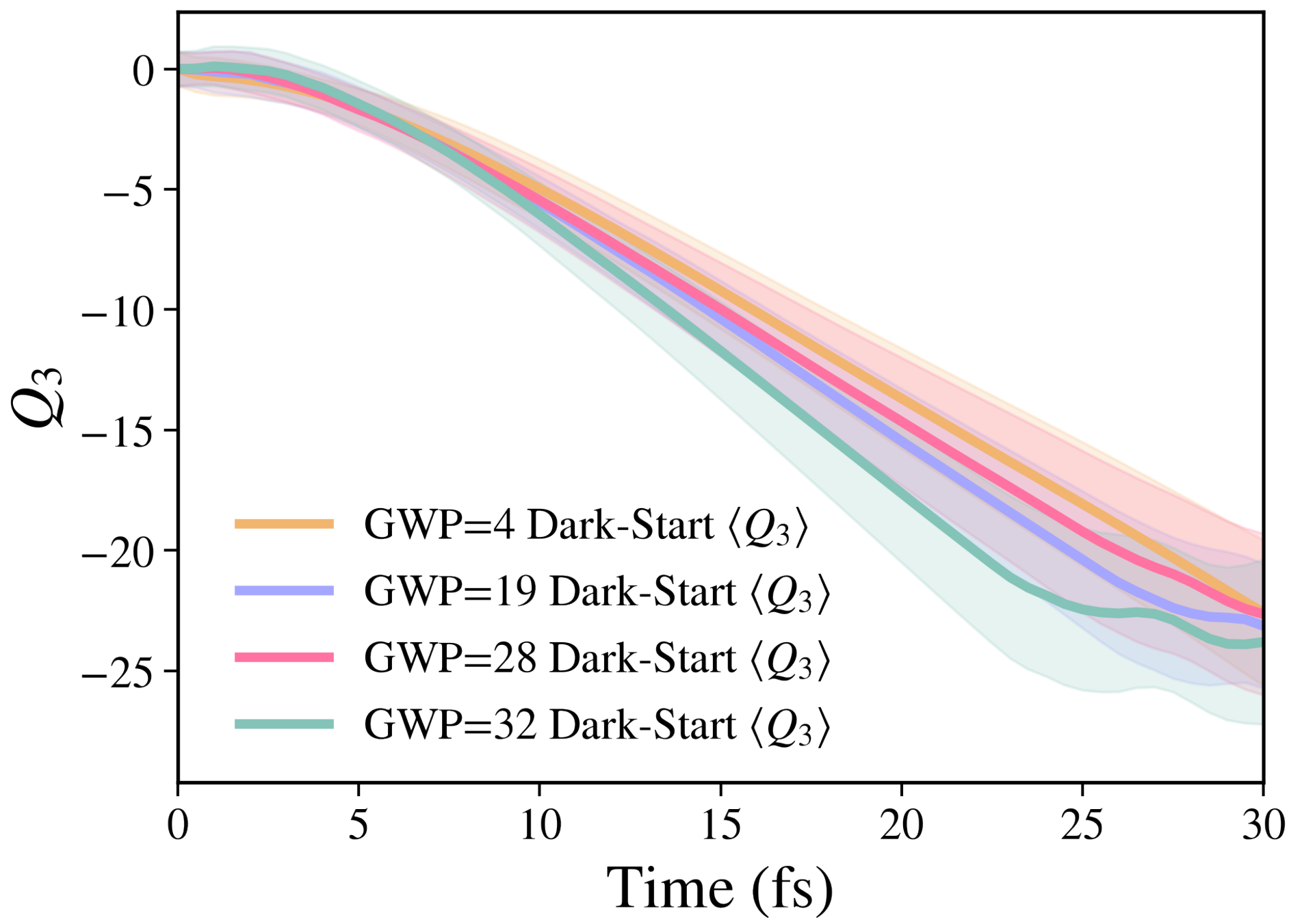

Figure 7: Expectation value $\langle Q_3 \rangle$ vs time, with shading extending coordinate width on either side, for the 4-GWP dark-start (orange), 19-GWP dark-start (blue), 28-GWP dark-start (red) and 32-GWP dark start (green). Decreasing Q_3 corresponds to O–O extension

7 State-resolved initial-database curvature

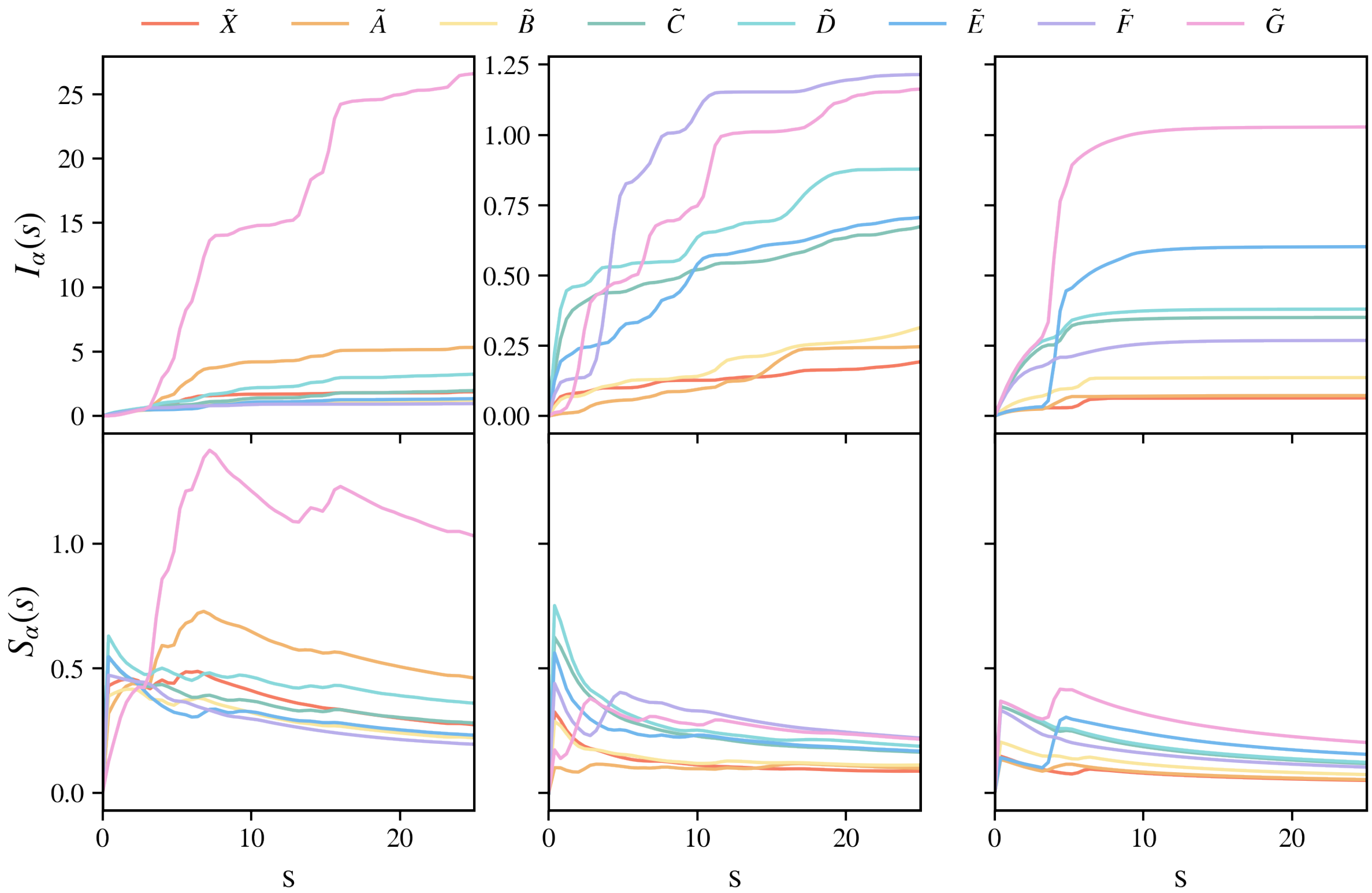

Figure 8: State-resolved accumulated squared curvature $I_\alpha(s)$ (top row) and running RMS curvature $S_\alpha(s)$ (bottom row) for three initial quasi-diabatic Hamiltonians. Left: deliberately sparse $N_G = 4$ *dark-start* initialisation. Centre: matched $N_G = 32$ *dark-start* initialisation. Right: initial *informed-sampling* database containing the FC entry and 97 additional seed entries

. The coordinate $s = Q_3^{\mathrm{FC}} - Q_3$ measures displacement from the FC region towards O–O extension. Each coloured curve denotes one quasi-diabatic state. The $N_G = 4$ calculation is a sensitivity test rather than a matched estimate of the sampling-protocol effect.

8 Additional normal-coordinate cuts

Figures 9 and 10 show additional diagonal quasi-diabatic cuts from the final approximately 5000-point databases. Each cut varies one rectilinear mass- and frequency-weighted normal coordinate while holding all other coordinates fixed.

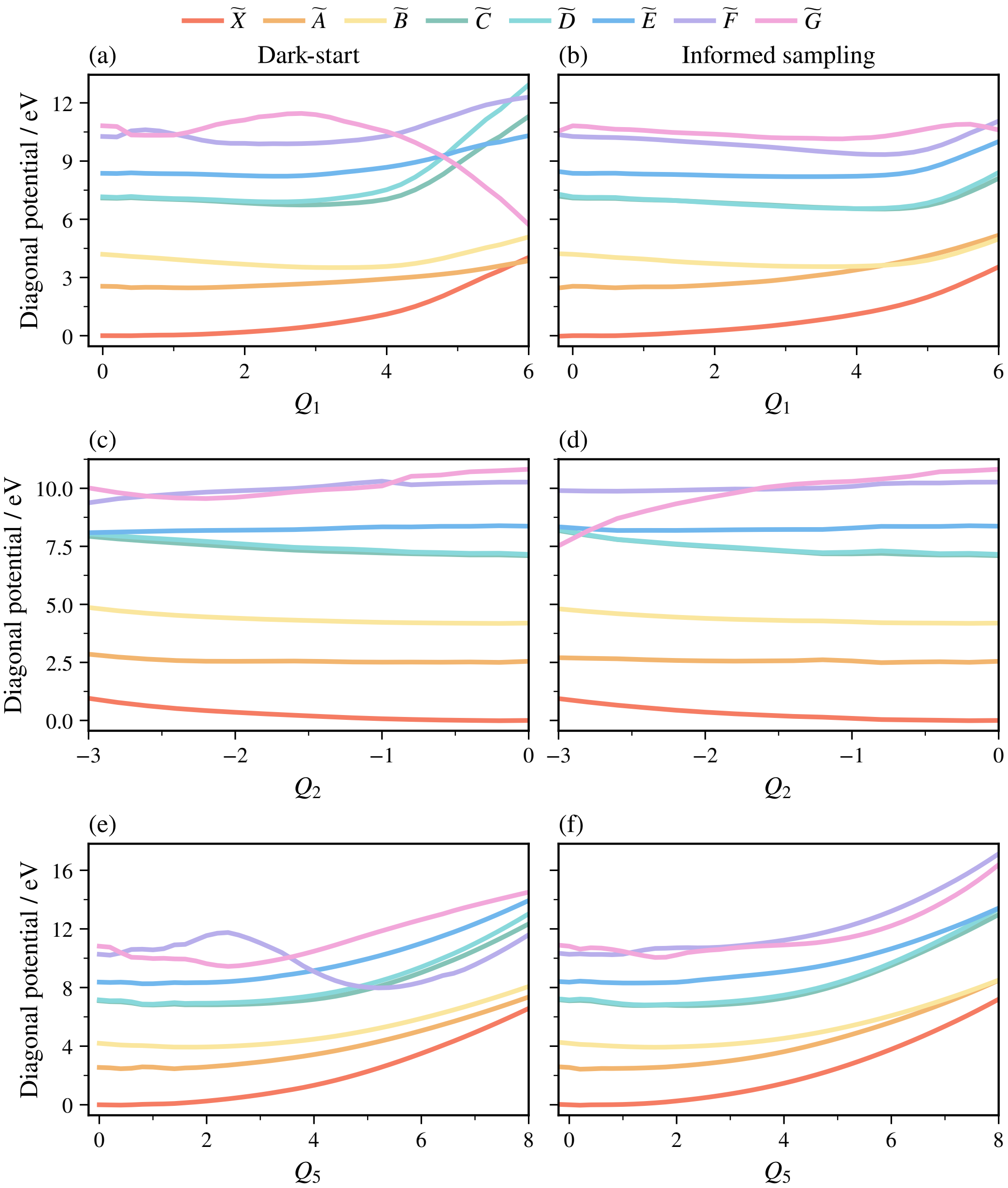

Figure 9: Diagonal quasi-diabatic potentials along Q_1 (a,b), Q_2 (c,d) and Q_5 (e,f), obtained from the final approximately 5000-point databases. The left column is dark-start and the right column is informed sampling. Only the selected mass- and frequency-weighted normal coordinate is varied; all others are held fixed. The eight fixed state colours identify $\tilde{X}, \tilde{A}, \ldots, \tilde{G}$ throughout. Each pair of panels uses the same coordinate and energy limits.

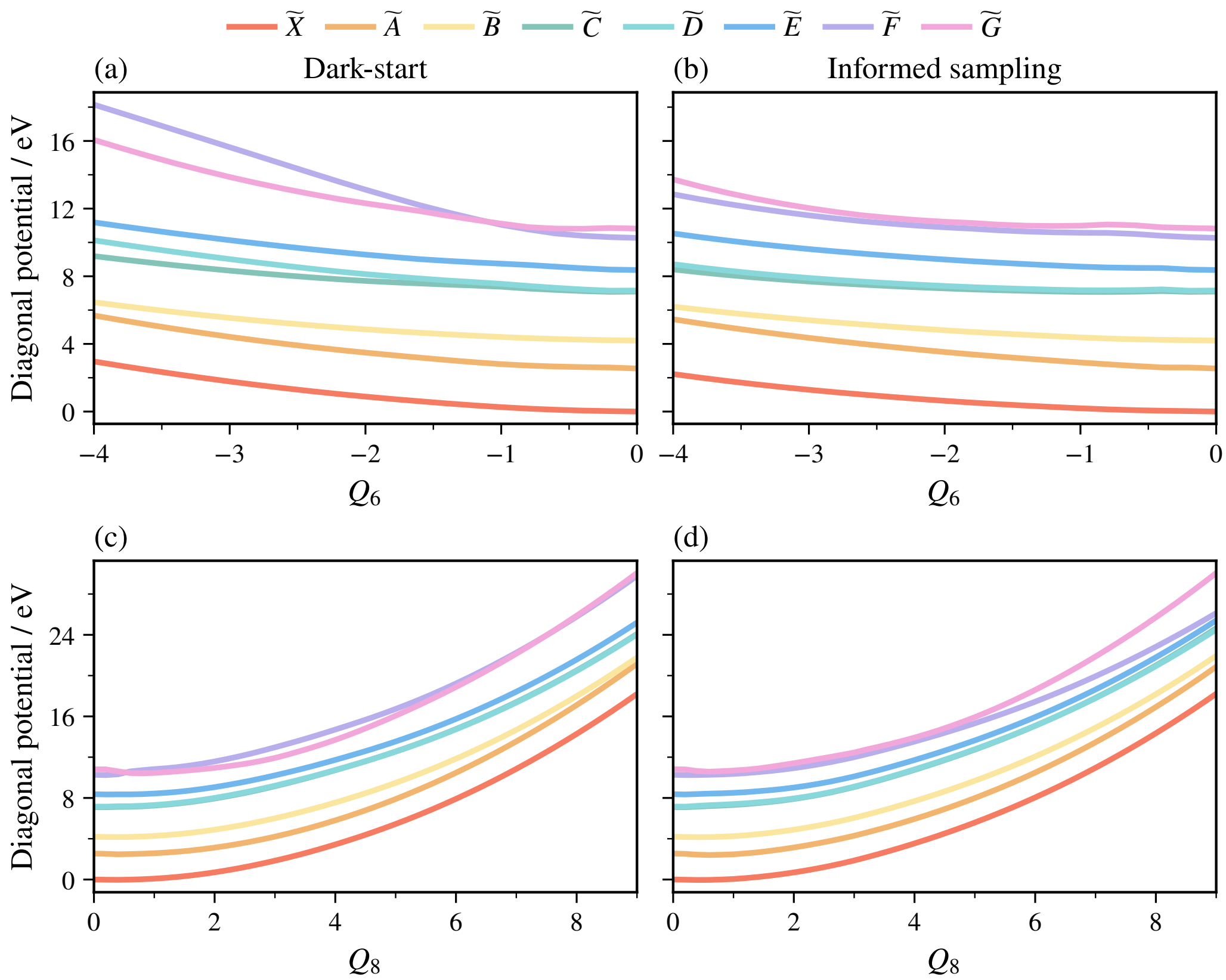

Figure 10: Diagonal quasi-diabatic potentials along Q_6 (a,b) and Q_8 (c,d), obtained from the final approximately 5000-point databases. The left column is dark-start and the right column is informed sampling. Only the selected mass- and frequency-weighted normal coordinate is varied; all others are held fixed. The eight fixed state colours identify $\tilde{X}, \tilde{A}, \ldots, \tilde{G}$ throughout. Each pair of panels uses the same coordinate and energy limits.

Table 1 applies the curvature diagnostic defined in Eqs. (13) and (14), with each state's squared curvature averaged over the complete common interval listed in the table. The differentiation and integration procedures are those described above. The two source grids coincide on each interval; unmatched endpoints are omitted without extrapolation. The informed values are lower on four of these cuts, but not on Q_2.

Table 1: Shared-grid aggregate RMS curvature, using source energies in eV and the plotted normal-coordinate units. All grids have spacing 0.2.

Coordinate	Common interval	Points	Dark-start	Informed sampling
Q_1	$[0, 6]$	31	8.1578	4.4372
Q_2	$[-3, 0]$	16	5.0849	5.5895
Q_5	$[0, 8]$	41	6.0147	5.2588
Q_6	$[-4, 0]$	21	4.3881	4.1247
Q_8	$[0, 9]$	46	4.6835	4.4327

9 Energy prediction and numerical propagation with read–write databases

A separate pair of read–write database propagations compares the workflows with 32 GWPs, eight electronic states, nine nuclear coordinates and the same adaptive RK5 accuracy setting of 10^{-6}. These calculations share the initial conditions and integration controls, but begin with different databases and subsequently request different geometries. During the first 20 fs the surviving probability remains above 0.9999 in both runs, allowing the numerical diagnostics to be compared before appreciable CAP absorption.

Figure 11(a) shows the reported energy drift $\Delta E(t) = E(t) - E(0)$. Its maximum absolute value over the 41 output samples at $0 \leq t \leq 20$ fs is 1.063263 eV for informed sampling and 2.006193 eV for dark-start. Dividing these full-wavefunction values by $N_G = 32$ gives the estimates 0.03323 and 0.06269 eV per GWP, respectively.

Over the post-startup interval 0.5–20 fs, informed sampling requires 1172 attempted RK5 steps, compared with 2148 for dark-start. Here an attempt is defined as an adaptive trial step: an accepted attempt advances time, whereas a rejected attempt is repeated. Step counts are assigned by their outer-interval boundaries (retaining the 0.5-fs intervals ending after 0.5 fs and at or before 20 fs.)

For the electronic-energy comparison, the conventional eight-state RMSE at each successfully evaluated geometry is

$$\mathrm{RMSE}_{\mathrm{states}} = \left[\frac{1}{8}\sum_{\alpha=1}^{8}\left(E_{\alpha}^{\mathrm{model}} - E_{\alpha}^{\mathrm{QC}}\right)^{2}\right]^{1/2}. \tag{22}$$

Table 2 reports statistics over each workflow's successful propagation queries at $0 \leq t < 20$ fs. Initial rediabatisation of existing database records is listed separately. The initial reference record in each workflow has no reported RMSE, and one dark-start propagation query failed in the electronic-structure calculation. These records are excluded from the statistics. Neither comparison is a same-geometry held-out test or a test of nonadiabatic couplings.

Table 2: Conventional eight-state energy RMSE statistics (eV). Propagation rows use successful queries at $0 \leq t < 20$ fs; initial rows use existing records processed before propagation. n counts valid RMSE values in each distinct sample set.

Stage	Workflow	n	Mean	Median	Maximum
Propagation	Informed sampling	522	0.062998	0.050479	0.316163
Propagation	Dark-start	571	0.076581	0.063904	0.477319
Initial	Informed sampling	3851	0.083460	0.056819	1.250856
Initial	Dark-start	4299	0.089135	0.066437	0.985229

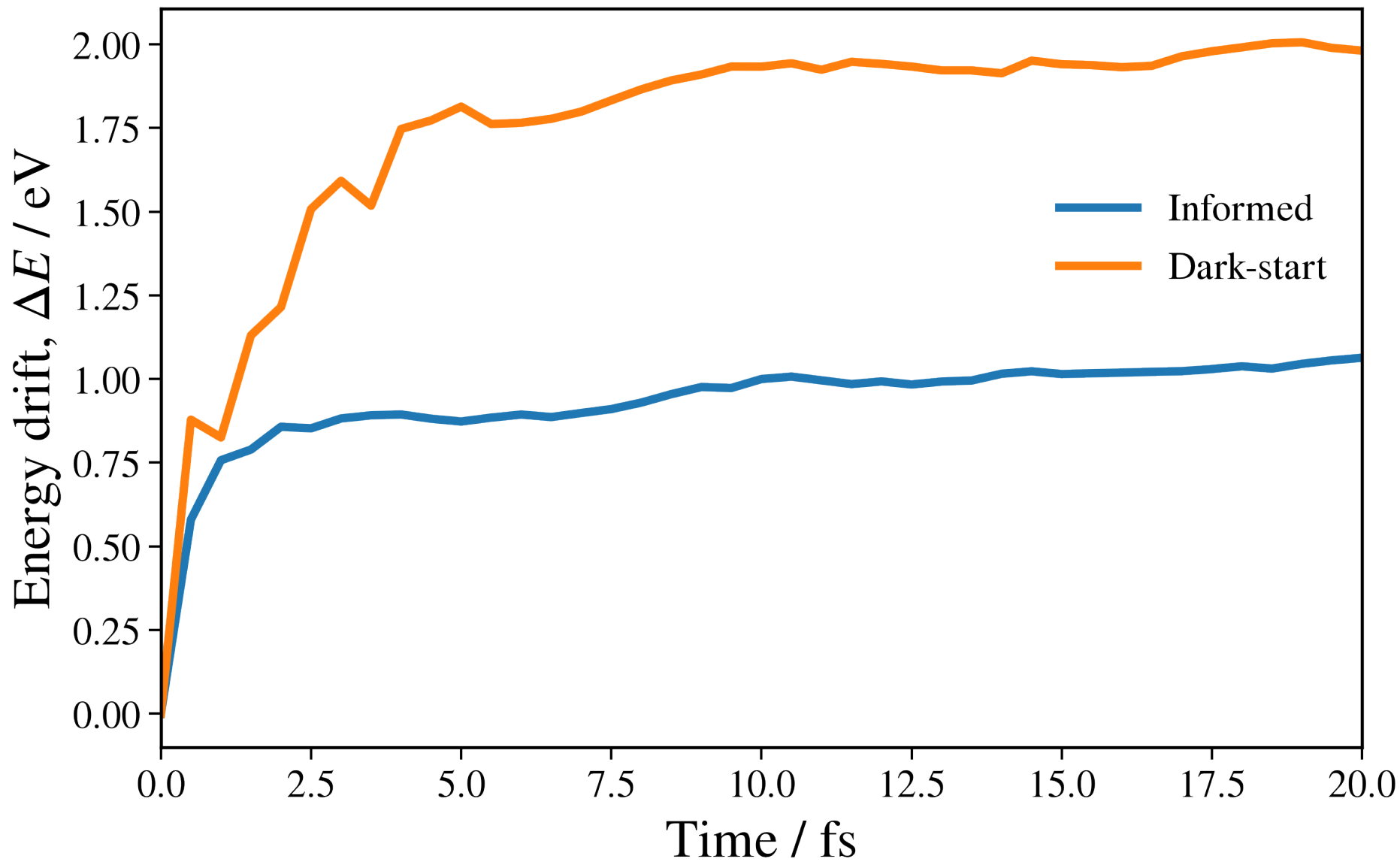

(a)

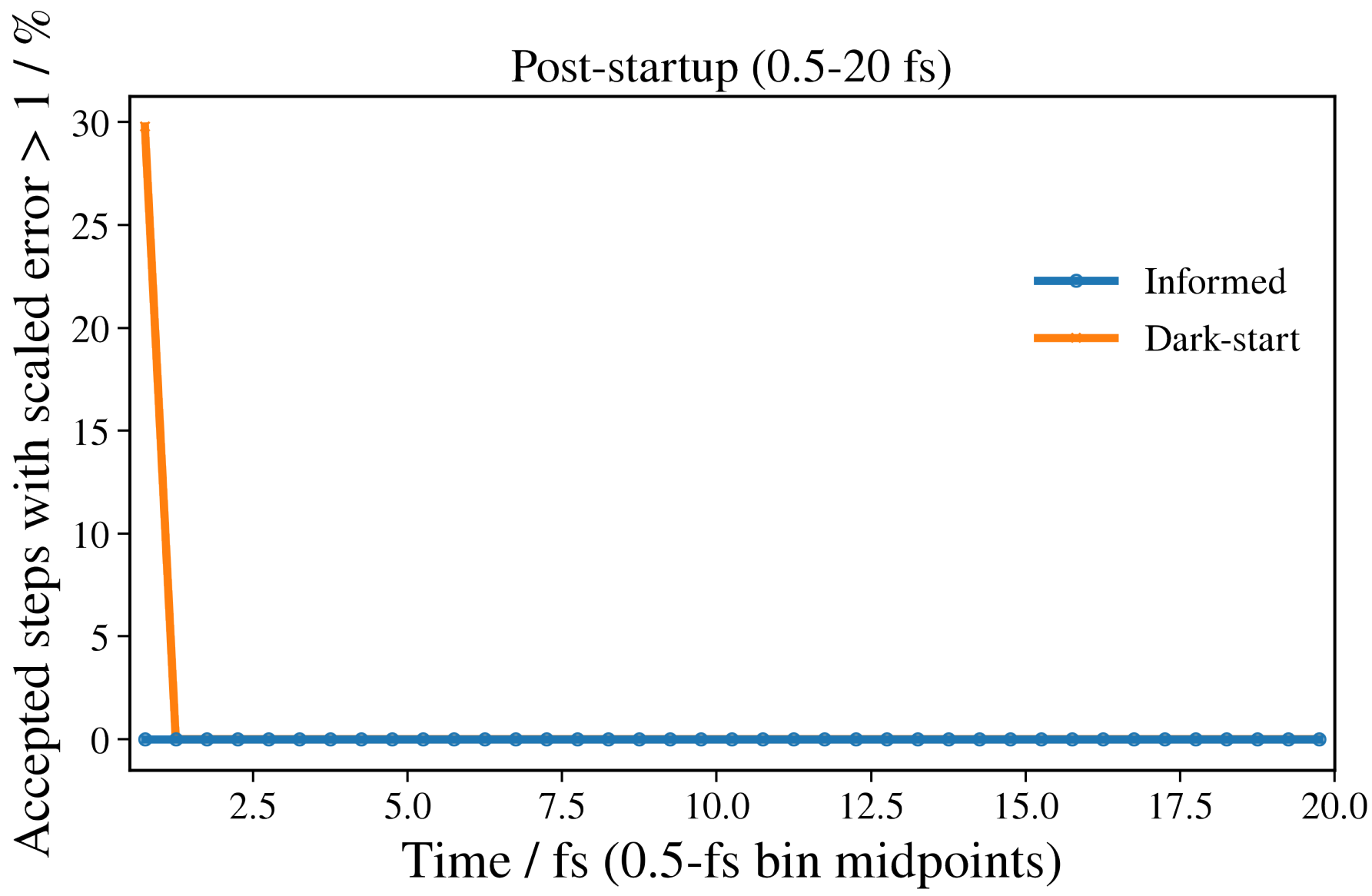

(b)

Figure 11: Numerical diagnostics for the read–write database propagations. (a) Reported energy drift through 20 fs, before appreciable CAP absorption. (b) Percentage of accepted RK5 steps with printed scaled error greater than one in each 0.5-fs interval over 0.5–20 fs, plotted at interval midpoints. The denominator is the number of accepted steps (attempted minus rejected). At the minimum step size of 10^{-4} fs, the integrator may accept a step even when its scaled error exceeds one.

10 Spectral-envelope comparison

Figure 12 compares spectral profiles from the complex wavepacket autocorrelations with the gas-phase absorption measurements tabulated by Ting et al. (Table S3)[5] and Mir et al. (Table S1).[6] The selected spectral exports are separate from the numerical-diagnostic pair in Sec. 9.

The profiles were evaluated with the Quantics `autospec` program using the command

```
autospec -f auto -lin -o EP-informed-lin.pl -EP -n -p 2000 2.6 7 ev
```

The same command was used for dark-start with the corresponding output filename. The energy grid contains 2001 points from 2.6 to 7.0 eV. The selected g2 profile is the fifth physical output column for these `-lin` exports. Both the real and imaginary parts of the stored autocorrelation $C(t)$ are retained. Apart from common constants, the energy-weighted profile is

$$S_{2,\mathrm{EP}}(E) \propto E \ \mathrm{Re} \int_0^T C(t) e^{iEt/\hbar} \cos^2\left(\frac{\pi t}{2T}\right) dt, \tag{23}$$

where T is the last autocorrelation time included from each input. The g2 cosine-squared taper is used without an additional exponential or Gaussian damping factor. The leading E is the `-EP` energy prefactor. The `-n` option peak-normalises each exported profile.

Positive energies are converted to wavelength using $\lambda[\mathrm{nm}] = 1239.841984332/E[\mathrm{eV}]$. To align the maxima with Ting's tabulated maximum at 346.0 nm, the wavelength axes are translated by $+116.52$ nm for dark-start, $+90.16$ nm for informed sampling and $+4.30$ nm for Mir's data. These are display translations, not corrections to the energy reference. After linear interpolation onto a common 0.1-nm grid over 310–430 nm, without extrapolation, the RMS intensity differences from Ting's data are 0.216527 for dark-start and 0.134388 for informed sampling; the corresponding values relative to Mir's data are 0.163220 and 0.089084. Thus the informed profile has closer agreement with these peak-aligned envelopes, although both calculations retain excess long-wavelength intensity. This comparison assesses envelope shape, not absolute band positions or absorption strengths.

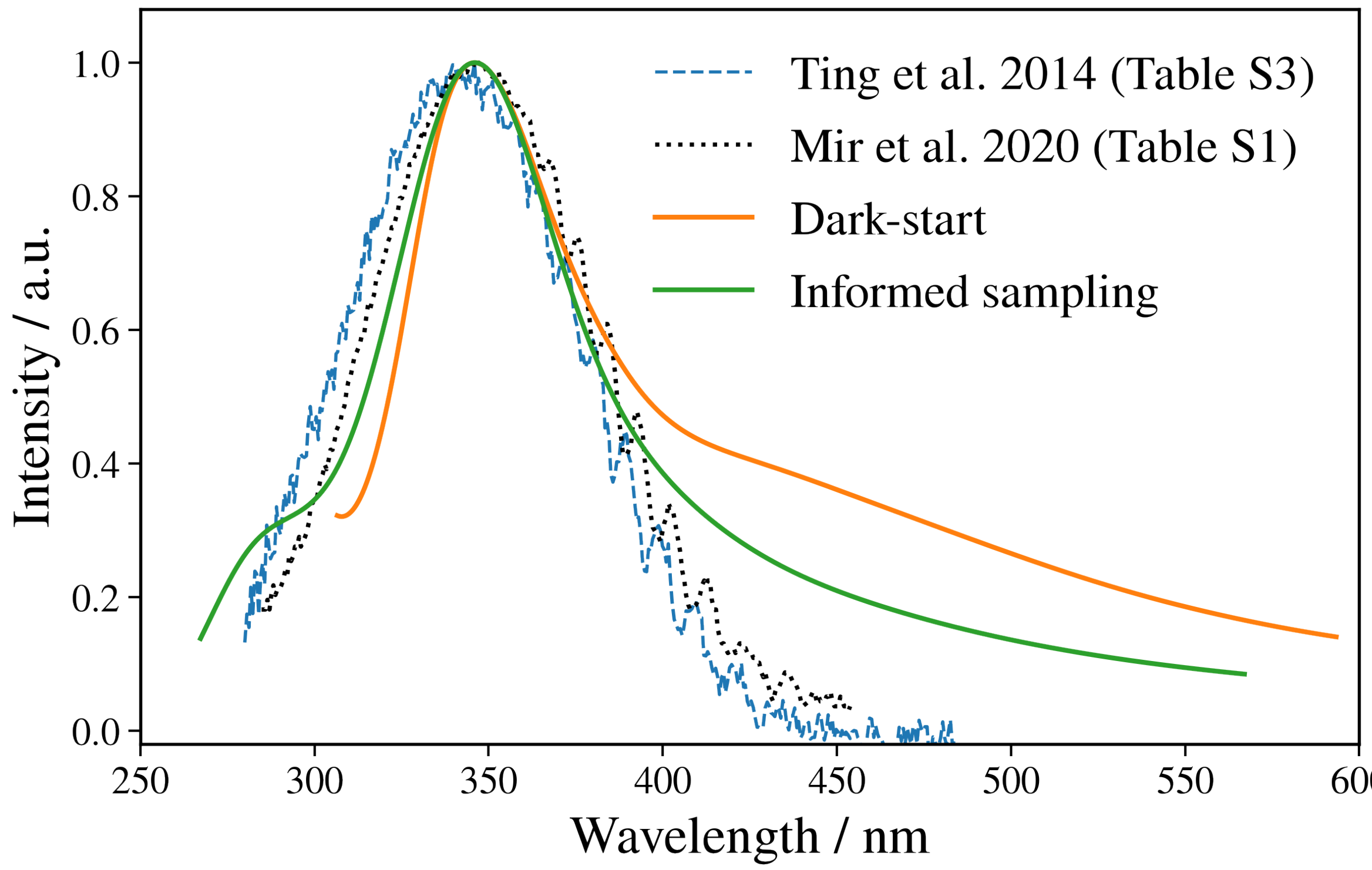

Figure 12: Peak-aligned spectral-envelope comparison with Ting et al. (2014, Table S3)[5] and Mir et al. (2020, Table S1).[6] Experimental curves are normalised to their own maxima. The wavelength translations are $+116.52$ nm (dark-start), $+90.16$ nm (informed sampling) and $+4.30$ nm (Mir), relative to Ting's 346.0-nm maximum. The comparison concerns envelope shapes rather than absolute spectral predictions.

11 Computational protocol and cost accounting

11.1 Database construction, Hessian updating and interpolation

The electronic-structure calculations used Molpro with the `nosym` and `noextra` keywords to turn off symmetry. All nine vibrational coordinates were retained in the dynamics without using any symmetry considerations. Explicit Hessians of all eight adiabatic potential energies were calculated only at the FC reference geometry. At the remaining seed geometries and geometries added during propagation, the electronic-structure calculations supplied adiabatic energies, their gradients and nonadiabatic coupling vectors. After construction of the ADT, these data were transformed into the quasi-diabatic representation. The quasi-diabatic Hessians entering the local harmonic models were obtained by Hessian updating using gradient information and previously available Hessians.[7, 8, 9]

Each database was accumulated through repeated 50 fs read–write propagations, retaining newly added entries between runs. Rediabatisation proceeded outwards from the FC reference. Observables and wavefunctions from the growth propagations were recorded at 0.5 fs intervals. The subsequent read-only population and nuclear-motion analyses used 0.1 fs output intervals. Settings for the separate read–write numerical-diagnostic pair are given in Sec. 9; the spectral inputs are identified separately in Sec. 10.

Where a new electronic-structure calculation was unnecessary, modified Shepard interpolation combined local quadratic expansions about nearby database geometries. For one quasi-diabatic potential-matrix element,

$$T_k(\boldsymbol{Q}) = W_k + \mathbf{g}_k^{\mathsf{T}} \Delta\boldsymbol{Q}_k + \frac{1}{2}\Delta\boldsymbol{Q}_k^{\mathsf{T}} \mathbf{H}_k \Delta\boldsymbol{Q}_k, \tag{24}$$

$$W_{\text{int}}(\boldsymbol{Q}) = \sum_k w_k(\boldsymbol{Q}) T_k(\boldsymbol{Q}), \qquad \sum_k w_k(\boldsymbol{Q}) = 1, \tag{25}$$

where $\Delta\boldsymbol{Q}_k = \boldsymbol{Q} - \boldsymbol{Q}_k$. Here W_k is the scalar value of the selected matrix element at database geometry k, $\mathbf{g}_k$ its coordinate-gradient vector, and $\mathbf{H}_k$ its coordinate-Hessian matrix. The weights favour nearby geometries. The interpolated potential and local derivative information supply the local harmonic approximation used for the Gaussian matrix elements.[8, 9]

11.2 Local cubic correction

Within each spin block, the diagonal elements of the stored quasi-diabatic potential matrix at the reference and of the predicted matrix at the new geometry are sorted separately, retaining their state-label indices. A difference between the resulting index sequences activates the cubic-correction branch. This branch is also activated when adjacent calculated adiabatic energies differ by less than 10^{-8} Hartree.

Let $\mathbf{R}_0$ and $\mathbf{R}_1$ denote the reference and new geometry in the coordinates used by the local database model. The length and unit direction of the straight segment are

$$L = \|\mathbf{R}_1 - \mathbf{R}_0\|, \qquad \hat{\mathbf{n}} = \frac{\mathbf{R}_1 - \mathbf{R}_0}{L}. \tag{26}$$

Along $\mathbf{R}(s) = \mathbf{R}_0 + s\hat{\mathbf{n}}$, the model is

$$\mathbf{W}(s) = \mathbf{C}_0 + \mathbf{C}_1 s + \mathbf{C}_2 s^2 + \mathbf{C}_3 s^3, \qquad 0 \le s \le L. \tag{27}$$

The constant, linear and quadratic coefficients are fixed by the reference quasi-diabatic potential, gradient and Hessian:

$$(C_0)_{ij} = W_{ij}(\mathbf{R}_0), \tag{28}$$

$$(C_1)_{ij} = \hat{\mathbf{n}} \cdot \nabla W_{ij}(\mathbf{R}_0), \tag{29}$$

$$(C_2)_{ij} = \frac{1}{2}\hat{\mathbf{n}}^{\mathsf{T}} \mathbf{H}_{ij}(\mathbf{R}_0)\hat{\mathbf{n}}. \tag{30}$$

Only the symmetric cubic coefficient matrix $\mathbf{C}_3$ is optimised.

At the endpoint, diagonalisation gives $\mathbf{U}^{\mathsf{T}}\mathbf{W}(L)\mathbf{U} = \mathrm{diag}(E_i^{\text{model}})$. The model directional-derivative matrix is

$$\mathbf{M}^{\text{model}} = \mathbf{U}^{\mathsf{T}}\mathbf{W}'(L)\mathbf{U}, \qquad \mathbf{W}'(L) = \mathbf{C}_1 + 2\mathbf{C}_2 L + 3\mathbf{C}_3 L^2. \tag{31}$$

The energy targets are the calculated adiabatic energies at $\mathbf{R}_1$. The derivative targets are

$$M_{ii}^{\text{target}} = \hat{\mathbf{n}} \cdot \nabla E_i, \qquad M_{ij}^{\text{target}} = \hat{\mathbf{n}} \cdot \mathbf{D}_{ij} \quad (i \neq j), \tag{32}$$

where $\mathbf{D}_{ij} = \langle\psi_i|\nabla\hat{H}_{\mathrm{el}}|\psi_j\rangle$ is the phase-aligned interstate-coupling vector. Away from exact degeneracy, $\mathbf{D}_{ij} = (E_j - E_i)\mathbf{F}_{ij}$.

The implemented objective combines the endpoint energy and directional-derivative residuals,

$$\epsilon = \left[\sum_i \left(E_i^{\mathrm{model}} - E_i^{\mathrm{target}}\right)^2 + \sum_{i\le j} \left(M_{ij}^{\mathrm{model}} - M_{ij}^{\mathrm{target}}\right)^2\right]^{1/2}, \tag{33}$$

evaluated in the program's internal units without additional weighting between these contributions. Diagonalisation of the optimised endpoint model supplies the ADT. That transformation is then applied to the calculated electronic-structure data to obtain the new quasi-diabatic database quantities.

11.3 Electronic-structure cost

The initial informed database comprises the common FC calculation and 97 additional path calculations. Table 3 separates the initial SA-CASSCF optimisation, gradient and nonadiabatic-coupling stages. Stage timings were estimated from differences between successive cumulative elapsed-time summaries in the Molpro outputs and averaged over the 97 additional seed calculations. The gradient and coupling stages include their associated CASSCF preparation, response-equation solutions and derivative evaluations. The FC calculation is reported separately.

The 97 additional jobs required a summed elapsed time of 40,337.71 s (11.2 h). Their mean complete-job time was 415.9 s (6.93 min). The common FC job required 15,700.71 s (4.36 h), giving 56,038.42 s (15.57 h) for all 98 initial entries. The FC calculation is required by both workflows and is therefore not included in the additional seeding overhead. These are sums of individual job times, not the elapsed duration of the sampling campaign.

Table 3: Electronic-structure stage timings. The non-FC column is the mean over the 97 additional seed jobs; the FC column gives the separate common FC job. Each job treats the complete eight-state manifold. The Hessian entry includes the associated frequency-analysis modules.

Stage	Non-FC mean / s	FC / s
Initial SA-CASSCF optimisation	13.0	7.15
Gradient response and eight state gradients	60.2	78.62
Coupling response and 28 pairwise NAC vectors	338.9	465.65
Explicit Hessians and frequency analysis	—	15144.14
Integral/orbital handling and other overhead	3.8	5.15
Complete job	415.9	15700.71

The archived calculations used the serial version of MOLPRO 2022.1, with one processor and 1000 MW of memory per processor.

Table 4 reports geometry-level electronic-structure calls during the four database-growth runs. Informed sampling required 3.40% fewer calls during growth. Including the 97 additional seed calculations and the common FC calculation gives 4053 and 4095 calls, respectively, a net reduction of 1.03%. These counts are distinct from the number of retained database entries and do not establish an equivalent reduction in elapsed computational time.

Table 4: Geometry-level electronic-structure calls for the two workflows. Growth-run counts exclude the initial seed and the common FC calculation. Internal evaluations within an electronic-structure job are not counted separately.

Stage	Informed sampling	Dark-start
Growth run 1	1022	1069
Growth run 2	991	986
Growth run 3	978	1072
Growth run 4	964	967
Growth total	3955	4094
Additional path calculations	97	0
Common FC calculation	1	1
Total	4053	4095

References

[1] Charlotte S. M. Allan et al. "A Straightforward Method of Analysis for Direct Quantum Dynamics: Application to the Photochemistry of a Model Cyanine". en. In: *J. Phys. Chem. A* 114.33 (Aug. 2010), pp. 8713–8729. ISSN: 1089-5639, 1520-5215. DOI: 10.1021/jp101574b. (Visited on 07/21/2026).

[2] G. A. Worth et al. *Quantics: A general purpose package for quantum dynamics simulations (version 2.2dev)*. Available from https://www.chem.ucl.ac.uk/quantics/. 2016.

[3] Harold W. Kuhn. "The Hungarian method for the assignment problem". In: *Naval Research Logistics Quarterly* 2 (1955), pp. 83–97.

[4] Pauli Virtanen et al. "SciPy 1.0: Fundamental Algorithms for Scientific Computing in Python". In: *Nat. Methods* 17 (2020), pp. 261–272. DOI: 10.1038/s41592-019-0686-2.

[5] Wei-Lun Ting et al. "The UV absorption spectrum of the simplest Criegee intermediate CH_2OO". In: *Phys. Chem. Chem. Phys.* 16 (2014), pp. 10438–10443. DOI: 10.1039/c4cp00877d.

[6] Zara S. Mir et al. "CH_2OO Criegee intermediate UV absorption cross-sections and kinetics of CH_2OO + CH_2OO and CH_2OO + I as a function of pressure". In: *Phys. Chem. Chem. Phys.* 22 (2020), pp. 9448–9459. DOI: 10.1039/D0CP00988A.

[7] T. J. Frankcombe. "Using Hessian update formulae to construct modified Shepard interpolated potential energy surfaces: Application to vibrating surface atoms". In: *J. Chem. Phys.* 140.11 (2014), p. 114108. DOI: 10.1063/1.4868637.

[8] G. W. Richings et al. "Quantum dynamics simulations using Gaussian wavepackets: the vMCG method". In: *Int. Rev. Phys. Chem.* 34 (2015), pp. 269–308. DOI: 10.1080/0144235X.2015.1051354.

[9] G. Christopoulou, A. Freibert, and G. A. Worth. "Improved algorithm for the direct dynamics variational multi-configurational Gaussian method". In: *J. Chem. Phys.* 154 (2021), p. 124127. DOI: 10.1063/5.0043720.
